# Supplementary material for: Barriers to implementation of enhanced recovery after surgery (ERAS) by a multidisciplinary team in China: a multicentre qualitative study
Source: BMJ Open. 2022 Mar 14;12(3):e053687. doi: 10.1136/bmjopen-2021-053687 (PMC8921855; doi:10.1136/bmjopen-2021-053687)
Supplement: Supplementary data [file bmjopen-2021-053687supp001.pdf]

Supplementary file 1 Detailed information on the implementation of ERAS in each center

Hospital	Year	Fields	Compliance
1	2015	Colorectal surgery, Gastrointestinal Surgery, Orthopedic surgery, Hepatobiliary surgery, Urological surgery, Thoracic surgery, Gynecological surgery, Otolaryngology surgery.	≥72%
2	2005	Colorectal surgery, Gastrointestinal Surgery , Orthopedic surgery, Hepatobiliary surgery, Urological surgery, Thoracic surgery, Gynecological surgery.	≥80%
3	2015	Hepatobiliary pancreatic surgery, Gastrointestinal Surgery, Thoracic surgery, Gynecological surgery, Urologic surgery.	≥90%
4	2015	Pancreatectomy, Pancreaticoduodenectomy.	≥80%
5	2012	Hepatobiliary pancreatic surgery, Gastrointestinal Surgery, Thoracic surgery, Urologic surgery.	≥85%
6	2010	Colorectal surgery, Gastrointestinal Surgery , Hepatobiliary surgery, Urological surgery, Thoracic surgery, Gynecological surgery.	≥90%

Each center implements the ERAS pathway in accordance with the ERAS guidelines.

Wang D, *et al. BMJ Open* 2022; 12:e053687. doi: 10.1136/bmjopen-2021-053687

Supplementary file 2 Institutional protocol

多中心研究协议书

项目名称： ERAS 模式下医患协同效果综合评价模型构建与推进路径研究

牵头单位：四川大学华西医院　　　研究负责人：李卡

ERAS 模式下医患协同效果综合评价模型构建与推进路径研究各参研中心就该研究相关事宜达成以下协议：

【1】 本单位严格遵守《赫尔辛基》宣言。

【2】 本单位对该研究的研究目的、研究方案、研究核心技术已经充分理解。

【3】 本单位对自身技术力量和工作时间安排经过慎重考虑后自愿参加该项协作研究。

【4】 本单位负责人对所提供的研究数据的真实性和准确性承担全部责任。

【5】 本单位接受和配合本研究学术委员会的监督和管理。

【6】 本单位如有学术不端行为（数据不实、伪造数据、抄袭、剽窃、重复发表、二级发表、一稿两投等），由本分中心负责人承担全部道德和法律责任，而研究主中心和其他分中心免责。

【7】 本单位认可并服从下述知识产权分配制度（主要指发表论文署名排序）的约定，具体如下：

1) 发表论文署名分配策略：（1）论文撰写者+（2）数据分析负责人+（3）有效病例数最多分中心单位的负责人+有效病例数第二多分中心单位的负责人+ ••• +有效病例数最少分中心单位的负责人+（4）其余参研人员（按提供有效病例数多少排序）+（5）本研究牵头 PI 作为通讯作者
2) 若杂志允许，则将论文撰写者、数据分析负责人及有效病例数最多分中心单位的负责人列为共同第一作者或共同通讯作者。
3) 对于期刊论文发表的特殊情况，作者署名尚需根据拟投杂志的要求进行相应调整。如某些 SCI 期刊严格限制论文署名作者数量不超过 8 名，因此仅能将贡献最大前 7-8 名作者列出，其余协作研究者列于文中致谢部分。
4) 为尽量全部体现参研人员的辛勤工作业绩，投稿选择期刊时尽可能避免有作者数量限制的期刊。
5) 期刊选择权及投稿权利在牵头 PI 单位。
6) 本研究相关结果的论文撰写及发表，由牵头 PI 单位执笔。

【8】 针对本研究收集的整体数据，如有参研单位负责人需要二次分析或其他研究目的进行分析时，需得到牵头 PI 单位的同意；参研单位负责人在学会发言需要使用本研究数据时，需注明数据来源并告知牵头 PI 单位。

【9】 参研单位的单中心数据，研究单位负责人有权自行保管，但需遵循隐私保护原则；对于发表的单中心数据结果、形式、内容，相关责任由发表中心负责人自行承担；单中心数据使用时，需告知数据中心并得到准确性认可：但是统计分析的单中心数据需注明来源，避免系统分析时重复纳入。

【10】 各单位数据若合理独立发表，其知识产权归所属分中心所有，不受本研究项目和其他分中心限制，但需在总体研究数据发表之后进行。

【11】 保密原则一：未经该协作研究半数以上分中心同意，任何个人及单位不得向除学术以外任何媒体或个人泄露研究数据及相关信息。

【12】 研究数据仅供学术研究所用，任何人不得将病例信息用于本研究涉及之外的用途。

【13】 每位论文贡献者均可依照国家及单位相关规定独立使用论文的知识产权，用于晋职、晋级或报奖，无需向其他合作者声明。

【14】 本研究如遇学术争议，依照国内外指南或共识解决，学术委员会有最终裁决权利。

【15】 本研究如遇知识产权争议甚至侵权时，可考虑第三方机构或组织进行磋商调停，或者甚至必要的法律援助。当出现学术不端行为所导致的不良影响或结果时，由当事学术不端研究者或研究单位承担相应道德和法律的责任。

【16】 本协作研究协议书自签字同意之日起执行并产生其协议法律效益。

我已充分理解本协作研究协议书的所有内容， 同意 **（同意/不同意）并自愿参加本研究，遵守所约定的所有内容。**

研究分中心：四川大学华西医院

分中心负责人签名：杨婕

日期：2020 年 9 月 1 日

牵头单位：四川大学华西医院

负责人签名：[illegible]

日期：2020 年 9 月 1 日
