# Supplementary material for: Barriers to implementation of enhanced recovery after surgery (ERAS) by a multidisciplinary team in China: a multicentre qualitative study
Source: BMJ Open. 2022 Mar 14;12(3):e053687. doi: 10.1136/bmjopen-2021-053687 (PMC8921855; doi:10.1136/bmjopen-2021-053687)
Supplement: Author's manuscript [file bmjopen-2021-053687.draft_revisions.pdf]

BMJ Open is committed to open peer review. As part of this commitment we make the peer review history of every article we publish publicly available.

When an article is published we post the peer reviewers' comments and the authors' responses online. We also post the versions of the paper that were used during peer review. These are the versions that the peer review comments apply to.

The versions of the paper that follow are the versions that were submitted during the peer review process. They are not the versions of record or the final published versions. They should not be cited or distributed as the published version of this manuscript.

BMJ Open is an open access journal and the full, final, typeset and author-corrected version of record of the manuscript is available on our site with no access controls, subscription charges or pay-per-view fees (http://bmjopen.bmj.com).

If you have any questions on BMJ Open's open peer review process please email info.bmjopen@bmj.com

BMJ Open

Barriers to implementation of enhanced recovery after surgery (ERAS) by a multidisciplinary team in China: a multicentre qualitative study

Journal:	*BMJ Open*
Manuscript ID	bmjopen-2021-053687
Article Type:	Original research
Date Submitted by the Author:	24-May-2021
Complete List of Authors:	Wang, Dan; Sichuan University, West China School of Nursing<br>Liu, Zhenmi; Sichuan University, West China School of Public Health and West China Fourth Hospital<br>Zhou, Jing; The Second People's Hospital of Chengdu<br>Yang, Jie; Sichuan University West China Hospital, Department of Gastrointestinal Surgery<br>Chen, Xinrong; Sichuan University, West China School of Nursing<br>Chang, Chengting; Sichuan University, West China School of Nursing<br>Liu, Changqing; Sichuan University, West China School of Nursing<br>Li, Ka; Sichuan University, West China School of Nursing<br>Hu, Jiankun; Sichuan University West China Hospital, Department of Gastrointestinal Surgery
Keywords:	QUALITATIVE RESEARCH, Organisation of health services < HEALTH SERVICES ADMINISTRATION & MANAGEMENT, Quality in health care < HEALTH SERVICES ADMINISTRATION & MANAGEMENT

SCHOLARONE™
Manuscripts

BMJ

I, the Submitting Author has the right to grant and does grant on behalf of all authors of the Work (as defined in the below author licence), an exclusive licence and/or a non-exclusive licence for contributions from authors who are: i) UK Crown employees; ii) where BMJ has agreed a CC-BY licence shall apply, and/or iii) in accordance with the terms applicable for US Federal Government officers or employees acting as part of their official duties; on a worldwide, perpetual, irrevocable, royalty-free basis to BMJ Publishing Group Ltd ("BMJ") its licensees and where the relevant Journal is co-owned by BMJ to the co-owners of the Journal, to publish the Work in this journal and any other BMJ products and to exploit all rights, as set out in our licence.

The Submitting Author accepts and understands that any supply made under these terms is made by BMJ to the Submitting Author unless you are acting as an employee on behalf of your employer or a postgraduate student of an affiliated institution which is paying any applicable article publishing charge ("APC") for Open Access articles. Where the Submitting Author wishes to make the Work available on an Open Access basis (and intends to pay the relevant APC), the terms of reuse of such Open Access shall be governed by a Creative Commons licence – details of these licences and which Creative Commons licence will apply to this Work are set out in our licence referred to above.

Other than as permitted in any relevant BMJ Author's Self Archiving Policies, I confirm this Work has not been accepted for publication elsewhere, is not being considered for publication elsewhere and does not duplicate material already published. I confirm all authors consent to publication of this Work and authorise the granting of this licence.

For peer review only

Barriers to implementation of enhanced recovery after surgery (ERAS) by a multidisciplinary team in China: a multicentre qualitative study

Correspondence to Professor Ka Li; lika127@126.com; Dr Jiankun Hu; hujkwch@126.com

Dan Wang,[1] Zhenmi Liu,[2] Jing Zhou,[3] Jie Yang,[4] Xinrong Chen,[1] Chengting Chang,[1] Changqing Liu,[1] Ka Li,[1] Jiankun Hu[4]

Affiliation

1. West China School of Nursing, West China Hospital, Sichuan University, Chengdu, China.

2. West China School of Public Health, West China Hospital, Sichuan University, Chengdu, China.

3. The Second People's Hospital of Chengdu, Chengdu, China.

4. Department of Gastrointestinal Surgery, West China Hospital, Sichuan University, Chengdu, China

Word count 5581words.

Keywords Enhanced recovery after surgery (ERAS); Multidisciplinary team (MDT); Qualitative research; SPO model.

For peer review only

For peer review only - http://bmjopen.bmj.com/site/about/guidelines.xhtml

ABSTRACT

Objective To explore the attitudes and barriers encountered in the implementation of enhanced recovery after surgery (ERAS) in China from the perspective of multidisciplinary team members.

Design Based on Donabedian's structure-process-outcome (SPO) model, a multicentre qualitative study using semistructured interviews was conducted.

Setting From September 2020 to December 2020, the participants of this study were interviewed from six tertiary hospitals in Sichuan Province (n=3), Jiangsu Province (n=2) and Guangxi Province (n=1) in China.

Participants A total of 42 members, including surgeons (n=11), anaesthesiologists (n=10), surgical nurses(n=14) and dietitians(n=7) were interviewed.

Results Multidisciplinary team (MDT) members still face many barriers during the process of implementing ERAS. The findings revealed that a shortage of medical resources, a lack of policy support, and outdated concepts are barriers to the structural dimension. The lack of communication and collaboration among MDT members, doctor-patient collaboration, and personalized management are barriers to the process dimension. Low compliance and high medical costs are barriers to the outcome dimension.

Conclusions In general, participants believed that the current implementation of ERAS was still based on ideas more than reality. Identifying key elements of problems in the application and promotion of ERAS from the perspective of the multidisciplinary team will provide a basis for the establishment of a quality evaluation system of ERAS and will promote the successful application and implementation of ERAS in the future.

For peer review only

Strengths and limitations of this study

- This study is the first multicentre qualitative study of ERAS in China that explored the barriers encountered in the implementation and promotion of ERAS from the perspective of multidisciplinary team members.
- This study included dietitians as one of the research objects to analyse the views and opinions of dietitians about the implementation of ERAS.
- This study used the SPO model as theoretical support to analyse the barriers in the application process of ERAS from a multidimensional perspective.
- Convenience sampling could lead to potential sampling bias.
- There were potential hints and prejudices in questions raised and interviewer's explanations.

INTRODUCTION

The surgical stress response associated with major surgery describes fundamental metabolic changes that lead to increased catabolism, immunosuppression, free radical production, and hypercoagulable states.[1] Enhanced recovery after surgery (ERAS) programs were introduced and implemented by Danish surgeon H. Kehlet in the 1990s.[1] The core concept of ERAS is to apply a series of evidence-based, innovative and optimized perioperative measures through multidisciplinary teamwork, such as prehabilitation, minimally invasive surgery, removal of invasive lines, multimodal analgesia, early mobilization and early oral feeding, to reduce ineffective and unhelpful medical interventions and reduce the incidence of postoperative stress and complications, thereby speeding up the recovery and adaptation of patients after surgery.[2-5] Based on these advantages, the ERAS program has been adopted internationally and applied widely in the field of surgery.

In China, the ERAS program was first proposed and implemented by Professor Jieshou Li in many fields of general surgery, such as stomach and colorectal surgery, and achieved a good curative effect for patients.[6 7] Subsequently, ERAS programs have

For peer review only - http://bmjopen.bmj.com/site/about/guidelines.xhtml

been popularized and applied in tertiary hospitals (the highest level) in various provinces of China for more than 10 years. Through a large number of randomized controlled trials (RCTs), it has been proven that the implementation of ERAS can effectively reduce the pain of patients, hospitalization costs and length of stay in the hospital without increasing the 30-day readmission rate and complications.[8-11] At present, the ERAS program has been successfully applied to general,[12] orthopaedic,[10] urological[11] and other operations,[13] and many expert consensuses have been developed for each specialty, marking the maturity of the application of ERAS in China.

There is currently sufficient evidence to support the hypothesis that the implementation of ERAS can indeed effectively improve the efficiency of the use of health service resources[14] and improve patient satisfaction with the diagnosis and treatment results. However, there are gaps in the importance and understanding of ERAS due to different regions, hospitals at different levels and experts in different disciplines, which limits the application and popularization of ERAS and causes the overall implementation of ERAS in China to be slow.

The ERAS pathway is carried out in the form of multidisciplinary cooperation, including multiple modes of surgery, anaesthesia, nursing and perioperative management to ensure the safety and effectiveness of patients during the perioperative period. [4] Previous studies have proven that multidisciplinary teams (MDTs) are an important guarantee for ERAS, and the effective cooperation of multidisciplinary teams is a key positive factor in promoting the application and advancement of ERAS.[15] Overall, the establishment of a patient-centred MDT is an inevitable trend in the development of ERAS. However, the construction and management of ERAS-MDT in China is still in the exploratory stage, and multidisciplinary members still face many difficulties and barriers in the various processes of implementing ERAS. Therefore, a targeted analysis of various barriers and a quality management plan are crucial to the construction and development of ERAS-MDT, which is expected to speed up the implementation of ERAS in China.

For peer review only - http://bmjopen.bmj.com/site/about/guidelines.xhtml

Previous studies have found many barriers to interprofessional cooperation among ERAS-MDTs, such as a lack of personnel, resistance to changes in traditional surgical concepts, poor patient compliance and collaboration, and a lack of unified management and operative plans for MDTs.[16-19] However, the current qualitative research mainly focused on surgeons, nurses, and anaesthesiologists. There are only limited data on the barriers encountered by dietitians in the implementation of ERAS, warranting further study.

In this study, based on Donabedian's structure-process-outcome health care quality model (SPO model), we presented the results of a qualitative study that aimed to gain an in-depth understanding of the views and opinions of multidisciplinary members in different regions on the implementation and promotion of the ERAS program and to explore the barriers that affect the effective implementation of ERAS, so as to provide a basis for the establishment of the quality evaluation system of ERAS, which can contribute to the development of ERAS programs in China.

METHODS

Based on the SPO model, we conducted a multicentre qualitative interview study to explore the experience of surgeons, anaesthesiologists, nurses and dietitians in implementing the ERAS program from the three aspects of structure, process and outcome and to analyse the barriers that affect the promotion and application of ERAS programs.

Design

We used a qualitative interview study underpinned by Donabedian's SPO model for health care quality in our study. Data were collected using semistructured interviews. In the SPO model, the 'structure' refers to the methods of resource allocation and the efficiency of resource utilization in medical services, such as the adequacy of facilities and equipment, sufficient staff and financial resources, and complete management

For peer review only - http://bmjopen.bmj.com/site/about/guidelines.xhtml

policies. ‘Process’ refers to the specific execution quantity and quality of each link in the implementation of medical activities according to the plan, such as the execution rate and execution quality of each ERAS program. The ‘outcome’ refers to the final result after the end of medical services, such as patient satisfaction, rehabilitation effect and medical cost. Although Donabedian proposed that the relationship among structure, process and outcome indicators is complex, it is essential for effective evaluation of medical quality.[20 21]

Setting

The ERAS program was mainly implemented in tertiary public hospitals in China, and convenience sampling was used to select hospitals in our study. The criteria for selecting the hospitals are shown in Table 1. Participants in our study were interviewed from six tertiary hospitals in Sichuan Province (n=3), Jiangsu Province (n=2) and Guangxi Province (n=1).

**Table 1** A summary of the criteria for selecting hospitals：
➢ A tertiary public hospital.
➢ A general hospital or a specialist hospital.
➢ The hospital has been implementing an ERAS program for at least 5 years.
➢ The hospital agreed to participate in this study.
➢ According to the principle of data saturation, sampling was stopped when there were no new codes or themes.

Sample

Purposive sampling was used to provide a diverse range of ages, genders, occupational classification and locations (Table 2).[22] A total of 42 people, including 11 surgeons, 10 anaesthesiologists, 14 surgical nurses and 7 dietitians, were interviewed. The inclusion criteria of the ERAS-MDT members were as follows: (1) Experience in ERAS

For peer review only

implementation; (2) More than 10 years of working experience; and (3) Gave informed consent and volunteered to participate in the study. Participants who could not express their ideas accurately and fully were excluded.

Table 2 Characteristics of interviewees

Mean age (SD, range)	37.36 (SD 5.94; range 28–52)
Characteristic	n(%)
Gender	
Male	20(47.62)
Female	22(52.38)
Occupational classification	
surgeon	11(26.19)
anaesthesiologist	10(23.81)
surgical nurse	14(33.33)
dietitian	7(16.67)
Location	
Sichuan	25(59.52)
Jiangsu	10(23.81)
Guangxi	7(16.67)

According to the inclusion and exclusion criteria, the relevant persons-in-charge of each hospital selected the research subjects who met the purpose of this study and sent a message to invite these research subjects to participate in this study. If they agreed to participate and share their experience and views on the implementation of ERAS, the first author (Dan Wang) contacted them by telephone and registered them to participate in this study with written informed consent.

Data collection and analysis

For peer review only - http://bmjopen.bmj.com/site/about/guidelines.xhtml

The participants were interviewed individually in the period from September to December 2020. Participants in Chengdu, Sichuan Province, conducted face-to-face interviews in a quiet room at their hospitals. Participants from other provinces were interviewed by telephone. The principal investigator (Dan Wang) and research assistant (Jing Zhou, Xinrong Chen and Chengting Chang) conducted the interviews together. None of the participants had a close personal relationship with the interviewers, and all participants agreed to be audiotaped. Interviewers needed to confirm any vague information provided by the interviewees in a timely manner. The average interview time was 21.84 minutes. Finally, the researcher made a brief summary to determine whether there was any missing or additional information and asked the interviewees to fill in the general situation form at the end of the interview. The researcher undertook reflection after each interview to check whether the interview process was inappropriate or needed to be improved and to confirm whether the data had reached saturation and whether the interview process needed to continue.

The recorded interview was transcribed verbatim within 24 hours. Researchers repeated line-by-line reading of the transcripts. Following familiarization, open coding was done manually, with codes written on the margin of the printed transcript.[23] These codes were subsequently grouped according to the SPO model to identify all themes that emerged from the data. When no new codes or topics appeared while the data were analysed, the research content reached saturation, and researchers could stop collecting research data.[24] To ensure reliability, all of the interview materials were reviewed separately by two investigators (Dan Wang and Cheng-ting Chang), the results were compared, and any conflicts were resolved in a timely manner by discussion with a third investigator (Jing Zhou). The interviews and transcripts are the main data sources for analysis.

Ethical approval

For peer review only - http://bmjopen.bmj.com/site/about/guidelines.xhtml

This study was approved by the Ethics Committee of Biomedical Research (West China Hospital of Sichuan University) (No.2020-1038). Data were treated confidentially. Each participant was assigned a code before data analysis (e.g., Interview 2, Anaesthesia, Male), and therefore, personal identification was removed.

Patient and public involvement

No patients were involved in this study. The public were not involved in the development of the research questions, research design or outcome measures.

RESULTS

Structure

a. Medical resources

All participants described the shortage of medical resources as the main barrier to their implementation of the ERAS program, and the most common description was the pressure and challenges caused by the shortage of human resources. Due to the application of ERAS, many measures needed to be implemented, and to shorten the average length of stay and improve the bed turnover rate, doctors and nurses must take care of more patients with limited resources and time. This situation has increased their workload, affecting the implementation quality of some ERAS programs and affected patient rehabilitation. Therefore, some doctors did not advocate the implementation of ERAS.

> In the area of nursing care, the most important thing is the increase in workload, which may cause some difficulties in the implementation of some measures, especially in very busy situations. We have also optimized the implementation process of related ERAS measures, but the quality of implementation would still be affected when the workload was particularly heavy. (Interview 39, Nurse, Female)

For peer review only

If we performed nerve block, it would take 10-20 minutes to prepare the equipment and materials, but for some surgeons, he could complete a minor surgery at this time. He thought that getting a nerve block would delay his operation time, so I think this would be the biggest barrier to developing ERAS. Because there were too many patients in China, surgeons had to perform more operations in a limited time to maintain a normal turnover rate. (Interview 13, Anaesthesia, Male)

Many participants described that the lack of financial resources of the hospital led to the poor promotion of the ERAS program. The workload of doctors and nurses has increased, but hospital leaders have not increased their corresponding salaries and bonuses, which could not motivate the medical staff to implement the ERAS program. Participants believed that hospital managers should formulate incentive policies to financially support and encourage medical staff to implement the ERAS program, thereby promoting the application of ERAS.

However, in terms of ERAS, direct assessment or incentive measures for our entire team were still lacking in hospital management. This meant that our salary and bonuses did not change after implementing so many ERAS measures. (Interview 25, Dietician, Male)

In addition, due to the shortage of financial resources, the operating room equipment of many hospitals has been in short supply. Surgeons and anaesthesiologists in 3 hospitals all mentioned that the operating room of the hospital was not sufficient to provide more equipment and space to satisfy all patients who could carry out the ERAS program. Even during the operation, the insulation equipment was in short supply, so not every patient could use the insulation equipment.

First, the temperature and equipment in the operating room were not complete. Not every patient in our department routinely uses equipment such as insulation blankets and infusion fluid warming, and not every patient can be monitored for temperature. (Interview 14, Anaesthesia, Male)

For peer review only - http://bmjopen.bmj.com/site/about/guidelines.xhtml

If we have more operating rooms, our nerve block can be performed in the preparation room in advance, so the whole process will be smoother. (Interview 19, Anaesthesia, Female)

b. Policy support

At the hospital level, many hospitals have not formulated and introduced corresponding incentive policies and measures to guide department leaders and medical staff to actively implement ERAS, so many medical staff do not generate internal driving forces and are unwilling to implement ERAS plans.

Although the deputy dean is now leading the ERAS program, it has not formed a large-scale development. Er, I think hospitals can stimulate our enthusiasm for implementing ERAS through various policies. For example, policy advocacy and active support should be strengthened, and the clinical implementation effects and scientific research results of ERAS should be vigorously published. However, currently hospitals generally do not have such a policy to encourage us. (Interview 11, Anaesthesia, Male)

Some participants believed that the country should implement ERAS in both tertiary hospitals and basic-level hospitals. The ERAS program promoted by tertiary hospitals is generally not implemented in basic-level hospitals, and the disconnection of concepts cannot ensure the smooth implementation of ERAS in the entire treatment process of patients.

Many tertiary hospitals can complete ERAS, but after patients leave this hospital, they return to the community, family, or transfer to some basic-level hospitals for some treatment. Can these basic-level hospitals keep up with the level of implementation of ERAS in tertiary hospitals? Currently, basic-level hospitals

For peer review only - http://bmjopen.bmj.com/site/about/guidelines.xhtml

generally do not carry out ERAS programs. This is actually a big problem. (Interview 2, Nurse, Female)

In addition, there is no formal training course or mechanism for the ERAS program in China. Medical staff only learn ERAS through meetings, discussions in the department, and exchanges with other hospitals. This informal training does not have a unified ERAS implementation process, which leads to great differences in the implementation of ERAS in various hospitals, and it is impossible to compare and analyse the implementation effects of multicentre hospitals.

There is currently no standardized ERAS training. Standardized training means that there is unified and standardized learning content, and then after the training is over, a nationally certified and formal training certificate will be issued. If this can be realized in the future, ERAS can be implemented in a more unified and standardized way, which will facilitate our routine multicentre research. (Interview 35, Anaesthesia, Male)

c. Outdated concepts

Doctors are reluctant to try new concepts mainly because they are worried about the safety of patients. The doctor-patient relationship is more subtle in China. If doctors encounter problems in the process of implementing ERAS, they will face greater work pressure and professional crises. Doctors take patient safety as their first priority in any medical measures they take, so they dare not try ERAS lightly.

It is mainly the idea of surgeons. Many surgeons are based on their own practice standards and are reluctant to accept the various new concepts of ERAS. This is the most critical issue. (...) For example, the ERAS guidelines require drinking water for two hours after surgery, but many surgeons and anaesthesiologists are unwilling. (Interview 11, Anaesthesia, Male)

For peer review only - http://bmjopen.bmj.com/site/about/guidelines.xhtml

I know that some surgeons are very resistant. We require local anaesthesia and analgesia for wounds, but the surgeon believes that this will lead to liquefaction of the wound and increase the incidence of infection. Although we told him that there are many literature reports that this will not increase the incidence of wound infection, he is very stubborn, and it is difficult for us to communicate with him. (Interview 14, Anaesthesia, Male)

Process

a. Communication and collaboration among multidisciplinary members

Participants believed that ERAS was the optimization and integration of a series of perioperative measures, which required efficient and dedicated multidisciplinary teams to communicate and cooperate effectively to promote its application and promotion. However, the construction of ERAS-MDTs in China is still in the exploratory stage. Participants mentioned that the main difficulty affecting the implementation of the ERAS program was the communication and collaboration between multidisciplinary teams, which would not only have a negative impact on the treatment of patients but also bring challenges to the smooth implementation of ERAS.

The issue of multidisciplinary collaboration is still very important. In fact, hospital leaders have been talking about strengthening multidisciplinary cooperation, but now there are very few that truly realize multidisciplinary cooperation, and for teams that do well in multidisciplinary cooperation, they have not been encouraged and played a leading role. I think that to develop ERAS well, we must do a good job in multidisciplinary cooperation. (Interview 14, Anaesthesia, Male)

In my opinion, multidisciplinary cooperation is the greatest difficulty in promoting ERAS. When it involves multiple disciplines, the knowledge background of each discipline may be different, and the perspective of dealing with the problem may be different. Well, we also encountered such practical problems in the implementation.

For peer review only - http://bmjopen.bmj.com/site/about/guidelines.xhtml

For example, surgeons and dieticians wanted patients to drink carbohydrate drinks two hours before surgery, but anaesthesiologists thought there was a risk of reflux and aspiration, so they did not agree to implement. Therefore, if there is no consensus among various disciplines, there will be differences in the implementation of ERAS. (Interview 25, Dietician, Male)

Participants described that the continuous and effective communication and collaboration of the multidisciplinary team throughout the perioperative period played a vital role in the smooth progress of ERAS. They also emphasized that different disciplines must reach a consensus on the ERAS program and jointly discuss and formulate a series of standardized implementation standards and procedures to improve the overall efficiency of collaboration between ERAS-MDT and increase the success rate of ERAS implementation.

Um... I think we should reach a consensus on how to carry out multidisciplinary collaboration in ERAS, and formulate a corresponding process. For example, the division of labour in each discipline is clear, and there is a dedicated person to coordinate the work between multiple disciplines. I believe this will greatly improve the efficiency of ERAS implementation. (Interview 22, Dietician, Female)

b. Doctor-patient collaboration

Participants also mentioned the importance of doctor-patient collaboration and described situations that hinder the development of some ERAS programs due to the unsatisfactory process of doctor-patient collaboration (Table 3). In terms of preoperative education, doctors believed that because patients have a low degree of understanding and significant medical knowledge gaps, if the information conveyed to patients is not sufficient and clear, the patient will not be able to accurately understand the specific connotations of the doctor’s instructions, which will lead to deviation in the

For peer review only - http://bmjopen.bmj.com/site/about/guidelines.xhtml

process of cooperation with patients and hinder the normal development of some ERAS programs (Table 3, quote 1 a and b).

The medical staff mainly mentioned that patients would mistakenly remember the time of preoperative fasting, which would affect ERAS implementation and delay the operation time. Some patients who have undergone traditional surgery will question the ERAS concept of preoperative fasting, worrying about accidents during the operation (Table 3, quote 2). In terms of early oral feeding, the nurses mentioned that they must personally supervise the patients to make them eat on time. If the supervision is not timely, the patient will delay eating. Some patients will refuse to eat early if they feel unwell, or if they feel unwell after eating, they will be dissatisfied with the quality of ERAS (Table 3, quote 3).

Participants said that the traditional concept of patients was that they needed to lie in bed for a long time after surgery because they worried that early mobilization would affect wound healing and aggravate pain (Table 3, quote 4 a and b). All dietitians mentioned that due to the shortened length of hospital stay and the lack of a complete postoperative follow-up mechanism, they could only obtain some subjective indicators through the phone, which made them unable to objectively and accurately evaluate the implementation effect of the ERAS nutrition program. The doctors believed that due to the shortened length of hospital stay, the patient's later treatment cannot be effectively guaranteed, which would increase the dissatisfaction of the patient. If postoperative complications occurred and the patient was re-admitted to the hospital for treatment, it would intensify the doctor-patient contradiction (Table 3, quote 5 a and b).

Table 3 Illustrative quotes representative of theme 2 (all participant names have been changed to digital codes)

Theme	Programs	Quotes	
**Doctor-patient collaboration**	**Preoperative education**	1	Is there enough time to fully communicate with patients? Patients often complained that doctor were too busy, and they could not find doctors if they wanted to consult. So have patients been given

For peer review only

		enough information by staff in hospitals? This has always been an issue worthy of attention. (Interview 32, Surgeon, Male)<br>Sometimes we thought we had explained contents clearly to the patient, but in fact he might not truly understand them. For example, we encountered a situation where we asked the patient to skip meals, but he thought that he could not eat rice, so he ate noodles. This is what we need to pay special attention to in the communication between doctors and patients. (Interview 27, Nurse, Female)
**Preoperative fasting**	2	Patients should be encouraged to eat a light meal up until 6 hours, and consume clear fluids up until 2 hours, before initiation of anaesthesia. Patients with traditional surgery experience would worry about aspiration, so he would not eat anything. Some patients would forget the exact time of fasting or postpone the prescribed time. These situations will certainly create obstacles to the implementation of ERAS. (Interview 40, Dietician, Female)
**Early oral feeding**	3	Patients would not voluntarily go to eat early after surgery, so we must always supervise and remind them. Some patients were reluctant to eat carbohydrate nutrient solution, mainly because they were worried that the gastrointestinal tract would be uncomfortable after taking it. (Interview 6, Nurse, Female)<br>We encouraged patients to eat early after surgery, but if they had gastric bloating after eating, they would question our ERAS. (Interview 30, Surgeon, Male)
**Early mobilization**	4	The traditional Chinese concept is to lie in bed for a long time after surgery, which is difficult for patients to change. (Interview 20, Anaesthesia, Male)<br>We would tell patients who early mobilization could enhanced recovery, but they would question this measure, worrying whether

For peer review only - http://bmjopen.bmj.com/site/about/guidelines.xhtml

		it would make surgical wound dehiscence or exacerbate pain. (Interview 1, Nurse, Female)
**Postoperative follow-up**	5	We hope to get the evaluation of the nutritional management effect of patients throughout the perioperative period. However, for example, patients with gallstone surgery were only hospitalized for one day, which made it difficult for us to obtain objective indicators for postoperative nutritional assessment. There was no way to evaluate the nutritional effects of patients after they were discharged from the hospital. (Interview 23, Dietician, Female)<br>Early discharge caused another problem, that was, the medical care and security that patients received after discharge were not enough, and they would feel dissatisfied. Once complications occurred and patients were re-admitted to the hospital, patients' dissatisfaction would explode, and the doctor-patient conflict would become more intense, which would cause many mental pressure on doctors. (Interview 28, Surgeon, Male)

Participants emphasized that patients' trust and adequate education were the key factors to improve the effect of doctor-patient collaboration. Some staff believed that patients' trust in doctors' diagnostic and treatment skills was the cornerstone of the implementation of ERAS. Once patients questioned the doctor's skills, which led to poor compliance, ERAS programs would be difficult to implement. In addition, adequate education could promote patients' understanding of the diagnosis and treatment plan and further improve patient satisfaction and compliance.

In the implementation of ERAS, doctor-patient collaboration is also very important. My personal view is that the patient needs to trust 100% of the diagnosis and treatment made by the medical staff. The second is that medical staff need to do adequate education with patients, such as telling patients about the benefits that new concepts and measures can bring to him, encouraging him to accept changes, and

For peer review only

better cooperating with us in various tasks. This is a better state of doctor-patient collaboration. (Interview 6, Nurse, Female)

c. Individualized management

Surgeons and anaesthesiologists mentioned that there was a general lack of personalized perioperative management programs for ERAS. Due to great individual differences of patients, medical staff should fully consider the individual characteristics of each patient's condition when implementing the ERAS program and adopt individualized management plans. Otherwise, safety problems may occur for some patients with a complicated aetiology and many serious comorbidities, which will affect the implementation effect of ERAS.

In fact, some patients could not perform activities so early after surgery. For example, for elderly patients, the time when they could perform activities may be relatively late. We still have to apply this concept of ERAS in an individualized way. (Interview 23, Dietician, Female)

Individualized management is truly important. Because current consensuses of experts were standardized procedures applicable to the general public, there were still individual differences between patients. For example, although people with the same disease, age, weight, and height have the same operation method, anaesthesia and nursing plan, they may have different postoperative outcomes. (Interview 12, Anaesthesia, Male)

Outcome

a. Compliance

All participants believed that the compliance of patients and families was one of the important factors affecting the effectiveness of ERAS and that good compliance was an important prerequisite for ensuring the safety and effectiveness of treatment.

For peer review only - http://bmjopen.bmj.com/site/about/guidelines.xhtml

Participants mentioned that the poor compliance of some patients and their families would reduce the effectiveness of ERAS and even cause a waste of medical resources.

We routinely required patients to take analgesics after surgery, the purpose of which was to reduce the dosage of opioids, reduce the occurrence of complications, and promote the recovery of patients. Occasionally, patients thought that the pain could be tolerated, so they arbitrarily refused to take analgesics prescribed by doctors. However, their pain would increase during activities, which would inevitably affect the implementation of early mobilization and other programs. (Interview 7, Nurse, Female)

Some patients were unwilling to be discharged early for fear that treatment after discharge would not be guaranteed. To stay in the hospital for a few more days, patients and families might lie about their illness, which greatly affects the doctor's diagnosis. For example, the patient already felt less pain in the wound, but he or families still said it was in severe pain, so our doctors could not let him out of the hospital, which would lead to prolonged hospitalization and waste of resources. (Interview 8, Surgeon, Male)

b. Cost

Anaesthetists mentioned that to save money, patients with poor economic conditions would choose to endure pain and refuse to use nerve blocks or analgesic pumps. Nutritionists believed that due to the high price of commercial nutritional preparations and the fact that medical insurance does not reimburse this part of the cost, some patients would refuse to use nutritional preparations, leading to the lack of essential nutrients in the diet, which affects the postoperative nutritional treatment.

Patients were very satisfied with the analgesic effect of nerve block, but the cost would increase by approximately 500 yuan. If I told patients who they could voluntarily choose whether to use this anaesthesia, those patients with poor

For peer review only - http://bmjopen.bmj.com/site/about/guidelines.xhtml

financial conditions would refuse to do the nerve block. In addition, the cost of postoperative analgesia pumps was nearly 1,000 yuan, and there were some patients who would rather bear the pain than spend more money. (Interview 19, Anaesthesia, Male)

The price of nutritional preparations is very expensive, with a bottle cost of more than 80 yuan. Patients needed to use at least 5 bottles before and after surgery, which would cost more than 400 yuan, and medical insurance was not reimbursed for this cost. Some families face great financial pressure, and they refuse to use nutritional preparation. If their nutrients were not adequate, it would definitely affect their recovery. (Interview 21, Dietician, Male)

DISCUSSION

Based on SPO theory, we conducted a qualitative study on multidisciplinary ERAS members of six tertiary hospitals in three provinces in China. We coded and refined the theme from the three dimensions of structure-process-outcome and summarized eight main barriers. To our knowledge, this is the first multicentre qualitative article that analyses ERAS application barriers from the perspective of multidisciplinary members in China.

On the structural dimension, limited medical resources were not conducive to improving the quality of medical services of the ERAS program. Existing medical resources could not fully meet the medical service needs of surgical patients[18] and could not guarantee that every patient would enjoy the accessibility and convenience of medical service resources. In the reality of a heavy disease burden and overloaded work intensity, the shortage of human resources has become an important factor hindering the sustained, stable and rapid development of ERAS. Pearsall et al.[19] conducted interviews with 55 medical staff and believed that institutional barriers such as a lack of human resources and a lack of financial resources in hospitals were one of the

For peer review only - http://bmjopen.bmj.com/site/about/guidelines.xhtml

barriers commonly mentioned by the interviewees. New findings in our study were that there was a funding gap between government financial appropriations and the actual needs of hospitals, and the government and hospitals had insufficient financial support for medical and health resources of ERAS, resulting in insufficient reserves of beds, equipment, medicines and other materials. In addition, MDT members who implemented ERAS did not receive increased salaries or bonuses, which led to the slow application of ERAS in clinical departments and did not promote a large-scale development trend.

The medical treatment combination was one of China's important measures to deepen the reform of public hospitals, aiming to build a scientific and reasonable medical service system through the alliance and resource sharing of medical and health institutions at different levels. However, there is still a disconnection phenomenon in the understanding of ERAS at different levels of hospitals. The new concepts and new technologies of the ERAS program in primary hospitals are still in the exploratory stage, failing to keep up with the implementation of ERAS in tertiary hospitals, resulting in patients still receiving traditional treatments during recuperation in primary hospitals after discharge, which makes the application of ERAS appear inconsistent. In addition, as with other study results, our study also discovered the importance of training and education for the promotion of ERAS.[17 19]

However, unlike other studies, we found that the absence of a unified standardized ERAS course training mechanism in China has led to deviations in the understanding and mastery of ERAS among multidisciplinary members. According to different local contexts and circumstances, the implementation of the ERAS pathway is different. Participants emphasized that the establishment of standardized ERAS training courses would provide more scientific and standardized knowledge education and skills training, which would help medical staff to change their concepts and achieve better consistency. Our study suggested that policies should be issued at the national level to vigorously support and promote the development of ERAS, build and promote a standardized

For peer review only - http://bmjopen.bmj.com/site/about/guidelines.xhtml

training system for ERAS, improve the awareness and acceptance of ERAS among medical staff at all levels of medical units, update and unify new concepts in a timely manner, promote the construction of the ERAS medical consortium and continue to improve supporting policies to strengthen the service capabilities of medical and health institutions.

In the process dimension, participants from the 6 hospitals all mentioned that the lack of effective cooperation among multidisciplinary teams has always been a barrier that hinders the implementation of ERAS. Previous studies have proven that close multidisciplinary cooperation is an inevitable requirement in the development of ERAS,[25] which contributes to the clinical implementation, quality control and continuous improvement of ERAS. However, our study demonstrated that the existing ERAS-MDT model still faces many difficulties in clinical practice. The current model lacks a unified and reasonable overall structure and construction plan, so multidisciplinary members have not fully reached a consensus on ERAS. The communication, coordination, and perioperative connection of various links need to be further optimized. This was followed by the following reasons: the lack of standardized ERAS implementation standards and procedures, the unclear responsibilities and division of labour of ERAS-MDT members, and the lack of scientific management of the implementation path and collaborative operation of ERAS-MDT, which limited the in-depth application of ERAS.

In addition, our study showed that the lack of doctor-patient collaboration was also one of the barriers to the implementation of ERAS, which could appear in all aspects of the perioperative period, resulting in the ERAS program being unsuccessful. The reasons are related to patients' poor understanding and acceptance of ERAS, as well as ineffective communication between doctors and patients. Studies have confirmed that good doctor-patient collaboration is an important factor in promoting the effective, safe and efficient operation of the healthcare system.[26] Staff need to spend enough time providing diagnostic and treatment services for patients and clearly explain their

For peer review only - http://bmjopen.bmj.com/site/about/guidelines.xhtml

condition to patients, which is a favourable factor for enhancing the collaboration between staff and patients. Our study showed that patients' high trust in medical staff and adequate education by medical staff are the key factors to improving doctor-patient collaboration. Liu B et al.[13] believed that medical staff answered patients' doubts adequately, which was crucial for responding to the needs of patients during the entire hospital stay. In addition, individualized management of patients can improve the safety and effectiveness of the implementation of ERAS, which can alleviate patients' worries about the concept, measures and effects of ERAS so that they can trust the medical plans adopted by staff and actively participate in their treatment and recovery. In summary, taking the patient as the centre and relying on a multidisciplinary team to formulate a standardized, individualized, and continuous comprehensive diagnosis and treatment plan is essential for the implementation of ERAS on the process dimension.

On the outcome dimension, participants agreed that poor compliance of patients and families would cause the quality of ERAS implementation to decline. Previous studies have shown that increased compliance with ERAS programs would lead to better treatment outcomes and may have long-term benefits for survival.[16] Therefore, the compliance of patients and families to all ERAS programs is an important indicator to ensure the implementation effect of ERAS. Our study demonstrated that high costs were one of the main reasons for the poor compliance of patients and families. With the continuous process of medical reform, medical insurance has covered more than 95% of residents in China and has gradually become an important way to solve the problem of expensive medical treatment for residents. However, it is not free medical care and patients still have to pay part of the cost, which would make some patients with poor financial conditions resist some uncovered treatment measures, such as not eating nutritional preparations and refusing analgesic pumps, resulting in unsatisfactory implementation effects of ERAS.

With the SPO model as a theoretical support, this study is an innovative multicentre qualitative study of ERAS in China that explores the barriers encountered in the

For peer review only - http://bmjopen.bmj.com/site/about/guidelines.xhtml

implementation and promotion of ERAS from the perspective of multidisciplinary team members. In addition, this study included dietitians as one of the research objects to understand and analyse the views and opinions of dietitians on the implementation of ERAS to compensate for the limitations of previous studies. This study also has limitations. First, the survey results were limited by the self-reporting nature of the interviews. We tried our best to collect different levels of information in terms of age, gender, working years, professional title, and working region to compensate for the limitations of personal cognition. Second, there were potential hints and prejudices in both the questions raised and the guidance and explanations of the interviewer. We selected assistants who did not know ERAS to participate in the interview to avoid personal experience affecting the views of the research subjects. Finally, this study did not interview members of all disciplines involved in the implementation of ERAS, such as pharmacists and rehabilitation doctors. In the future, we will further expand the types of research objects.

Conclusions

This study analysed the barriers to the implementation of the ERAS program in the structure-process-outcome dimensions from the perspective of multidisciplinary members. In general, participants believed that the current implementation of ERAS was still based on ideas more than reality. The main barriers in the structure dimension were the shortage of medical resources, lack of policy support, and ingrained outdated concepts. In the process dimension, it was mainly the lack of communication and collaboration between multidisciplinary teams, the lack of doctor-patient collaboration, and the lack of individualized management plans. The outcome dimension was mainly low compliance and high medical costs. Identifying the key elements of the problems in the application and promotion of ERAS will help provide a basis for the establishment of a quality evaluation system for ERAS in the future and will promote the widespread successful application and implementation of ERAS.

For peer review only - http://bmjopen.bmj.com/site/about/guidelines.xhtml

Footnotes

Contributors KL planned the study. DW was principal investigator, collected, analysed and interpreted the data, and wrote the manuscript. KL, DW and ZML designed the study, developed the methods. ZML, CQL and JKH reviewed/edited the manuscript. JZ, CTC, XRC collected, analysed and checked the data. JY helped draft the paper, provided critical reviews and intellectual content. All authors have seen and approved the final version of the manuscript. DW is the guarantor. KL, ZML and JKH are on the supervisory team for DW PhD study.

Finding This work was supported by a program of National Natural Science Foundation of China (71974135). The funders had no role in the study design, data collection and analysis, decision to publish, or preparation of the manuscript.

Competing interests None declared.

Data availability statement Data are available upon reasonable request. The search strategies are available on request to the corresponding author.

Patient consent for publication Not required.

Ethics approval Ethical approval for this study was granted by the Ethics Committee of Biomedical Research (West China Hospital of Sichuan University) (No.2020-1038). Data was treated confidentially.

Provenance and peer review Not commissioned; externally peer reviewed.

REFERENCES

For peer review only - http://bmjopen.bmj.com/site/about/guidelines.xhtml

1. Kehlet H. Multimodal approach to control postoperative pathophysiology and rehabilitation. *Br J Anaesth* 1997;78:606-617.
2. Nelson G, Bakkum-Gamez J, Kalogera E, *et al*. Guidelines for perioperative care in gynecologic/oncology: Enhanced Recovery After Surgery (ERAS) Society recommendations-2019 update. *Int J Gynecol Cancer* 2019;29:651-668.
3. Wilmore DW, Kehlet H. Management of patients in fast track surgery. *Br Med J* 2001;322:473–476.
4. Ljungqvist O, Scott M, Fearon KC. Enhanced recovery after surgery: a review. *JAMA Surg* 2017;152:292-298.
5. Department of Health. Enhanced recovery partnership programme project report. 2011 https://www.gov.uk/government/publications/ enhanced-recovery-partnership-programme (accessed 1 Feb 2017).
6. Zhao J, Hu J, Jiang Z, *et al*. Outcome of Discharge Within 72 Hours of Robotic Gastrectomy Using Enhanced Recovery After Surgery Programs. *J Laparoendosc Adv Surg Tech A* 2018;28:1279-1286.
7. Liu XX, Pan HF, Jiang ZW, *et al*. "Fast-track" and "Minimally Invasive" Surgery for Gastric Cancer. *Chin Med J (Engl)* 2016;129:2294-2300.
8. Kjølhede P, Bergdahl O, Borendal Wodlin N, *et al*. Effect of intrathecal morphine and epidural analgesia on postoperative recovery after abdominal surgery for gynecologic malignancy: an open-label randomised trial. *BMJ Open* 2019;9:e024484.
9. Liang X, Ying H, Wang H, *et al*. Enhanced recovery care versus traditional care after laparoscopic liver resections: a randomized controlled trial. *Surg Endosc* 2018;32:2746-2757.
10. Cui H, Sun Z, Ruan J, *et al*. Effect of enhanced recovery after surgery (ERAS) pathway on the postoperative outcomes of elbow arthrolysis: A randomized controlled trial. *Int J Surg* 2019;68:78-84.

For peer review only - http://bmjopen.bmj.com/site/about/guidelines.xhtml

11. Lin T, Li K, Liu H, *et al*. Enhanced recovery after surgery for radical cystectomy with ileal urinary diversion: a multi-institutional, randomized, controlled trial from the Chinese bladder cancer consortium. *World J Urol* 2018;36:41-50.
12. Xu X, Wang Y, Feng T, *et al*. Nonstrict and individual enhanced recovery after surgery (ERAS) in partial hepatectomy. *Springerplus* 2016;5:2011.
13. Liu B, Liu S, Wang Y, *et al*. Neurosurgical enhanced recovery after surgery (ERAS) programme for elective craniotomies: are patients satisfied with their experiences? A quantitative and qualitative analysis. *BMJ Open* 2019;9:e028706.
14. Yamagata Y, Yoshikawa T, Yura M, *et al*. Current status of the "enhanced recovery after surgery" program in gastric cancer surgery. *Ann Gastroenterol Surg* 2019;3:231-238.
15. Paton F , Chambers D , Wilson P , *et al*. Effectiveness and implementation of enhanced recovery after surgery programmes: a rapid evidence synthesis. *BMJ Open* 2014;4:e005015.
16. Seow-En I, Wu J, Yang LWY, *et al*. Results of a colorectal enhanced recovery after surgery (ERAS) programme and a qualitative analysis of healthcare workers' perspectives. *Asian J Surg* 2021;44:307-312.
17. Cohen R, Gooberman-Hill R. Staff experiences of enhanced recovery after surgery: systematic review of qualitative studies. *BMJ Open* 2019;9:e022259.
18. Wong DJN, Harris SK, Moonesinghe SR, *et al*. Cancelled operations: a 7-day cohort study of planned adult inpatient surgery in 245 UK National Health Service hospitals. *Br J Anaesth* 2018;121:730-738.
19. Pearsall EA, Meghji Z, Pitzul KB, *et al*. A qualitative study to understand the barriers and enablers in implementing an enhanced recovery after surgery program. *Ann Surg* 2015;261:92-96.
20. Donabedian A. Evaluating the quality of medical care. 1966. *Milbank Q* 2005;83:691-729.

For peer review only - http://bmjopen.bmj.com/site/about/guidelines.xhtml

21. Donabedian A. The quality of care. How can it be assessed? *JAMA* 1988;260:1743-8.

22. Mays N, Pope C. Rigour and qualitative research. *BMJ* 1995;311:109–12.

23. Braun V, Clarke V. Using thematic analysis in psychology. *Qual Res Psychol* 2006;3:77–101.

24. Denzin NK, Lincoln YS. *The SAGE Handbook of qualitative research*. Fourth edn. Los Angeles: Sage Publications, 2011.

25. Li L, Jin JY, Min S, *et al*. Compliance with the enhanced recovery after surgery protocol and prognosis after colorectal cancer surgery: a prospective cohort study. *Oncotarget* 2017,8:53531-53541.

26. Penm J, MacKinnon NJ, Strakowski SM, *et al*. Minding the Gap: Factors Associated With Primary Care Coordination of Adults in 11 Countries. *Ann Fam Med* 2017;15:113-119.

For peer review only

For peer review only - http://bmjopen.bmj.com/site/about/guidelines.xhtml

Standards for Reporting Qualitative Research (SRQR)*

http://www.equator-network.org/reporting-guidelines/srqr/

Title and abstract

	Page/line no(s).
**Title** - Concise description of the nature and topic of the study Identifying the study as qualitative or indicating the approach (e.g., ethnography, grounded theory) or data collection methods (e.g., interview, focus group) is recommended	P1/1-3.
**Abstract** - Summary of key elements of the study using the abstract format of the intended publication; typically includes background, purpose, methods, results, and conclusions	P2/1-28.

Introduction

**Problem formulation** - Description and significance of the problem/phenomenon studied; review of relevant theory and empirical work; problem statement	P3/13-28, P4/1-28, P5/1-7.
**Purpose or research question** - Purpose of the study and specific objectives or questions	P5/8-14.

Methods

**Qualitative approach and research paradigm** - Qualitative approach (e.g., ethnography, grounded theory, case study, phenomenology, narrative research) and guiding theory if appropriate; identifying the research paradigm (e.g., postpositivist, constructivist/ interpretivist) is also recommended; rationale**	P5/16-28, P6/1-7.
**Researcher characteristics and reflexivity** - Researchers' characteristics that may influence the research, including personal attributes, qualifications/experience, relationship with participants, assumptions, and/or presuppositions; potential or actual interaction between researchers' characteristics and the research questions, approach, methods, results, and/or transferability	P6/17-21, P7/1-2.
**Context** - Setting/site and salient contextual factors; rationale**	P6/9-15.
**Sampling strategy** - How and why research participants, documents, or events were selected; criteria for deciding when no further sampling was necessary (e.g., sampling saturation); rationale**	P6/9-21, P7/1-8.
**Ethical issues pertaining to human subjects** - Documentation of approval by an appropriate ethics review board and participant consent, or explanation for lack thereof; other confidentiality and data security issues	P8/9-13.
**Data collection methods** - Types of data collected; details of data collection procedures including (as appropriate) start and stop dates of data collection and analysis, iterative process, triangulation of sources/methods, and modification of procedures in response to evolving study findings; rationale**	P7/10-24.

For peer review only - http://bmjopen.bmj.com/site/about/guidelines.xhtml

**Data collection instruments and technologies** - Description of instruments (e.g., interview guides, questionnaires) and devices (e.g., audio recorders) used for data collection; if/how the instrument(s) changed over the course of the study	P8/1-14.
**Units of study** - Number and relevant characteristics of participants, documents, or events included in the study; level of participation (could be reported in results)	P6/17-21, P7/1-5.
**Data processing** - Methods for processing data prior to and during analysis, including transcription, data entry, data management and security, verification of data integrity, data coding, and anonymization/de-identification of excerpts	P7/6-13, P8/1-11.
**Data analysis** - Process by which inferences, themes, etc., were identified and developed, including the researchers involved in data analysis; usually references a specific paradigm or approach; rationale**	P8/15-25.
**Techniques to enhance trustworthiness** - Techniques to enhance trustworthiness and credibility of data analysis (e.g., member checking, audit trail, triangulation); rationale**	P8/11-14.

Results/findings

**Synthesis and interpretation** - Main findings (e.g., interpretations, inferences, and themes); might include development of a theory or model, or integration with prior research or theory	P9/10-21, P10/7-13,19-24, P11/5-9,18-22, P12/4-9,17-22, P13/8-17, P14/7-13,21-28, P15/1-22, P16/1-7, P17/1-7, P18/4-11,24-28, P19/1-2,19-25.
**Links to empirical data** - Evidence (e.g., quotes, field notes, text excerpts, photographs) to substantiate analytic findings	P9/22-27, P10/1-6,25-28, P11/1-3,10-16,24-27, P12/1-2,10-15,23-27, P13/1-6,18-28, P14/15-19, P15/3, P16, P17/8-12, P18/1-2,12-22, P19/3-17,26-28, P20/1-11.

Discussion

**Integration with prior work, implications, transferability, and contribution(s) to the field -** Short summary of main findings; explanation of how findings and conclusions connect to, support, elaborate on, or challenge conclusions of earlier scholarship; discussion of scope of application/generalizability; identification of unique contribution(s) to scholarship in a discipline or field	P20/13-28, P21/1-28, P22/1-28, P23/1-28, P24/1-14.

For peer review only

**Limitations** - Trustworthiness and limitations of findings	P24/4-14.

Other

**Conflicts of interest** - Potential sources of influence or perceived influence on study conduct and conclusions; how these were managed	P25/14.
**Funding** - Sources of funding and other support; role of funders in data collection, interpretation, and reporting	P25/10-12.

*The authors created the SRQR by searching the literature to identify guidelines, reporting standards, and critical appraisal criteria for qualitative research; reviewing the reference lists of retrieved sources; and contacting experts to gain feedback. The SRQR aims to improve the transparency of all aspects of qualitative research by providing clear standards for reporting qualitative research.

**The rationale should briefly discuss the justification for choosing that theory, approach, method, or technique rather than other options available, the assumptions and limitations implicit in those choices, and how those choices influence study conclusions and transferability. As appropriate, the rationale for several items might be discussed together.

**Reference:**	
O'Brien BC, Harris IB, Beckman TJ, Reed DA, Cook DA. **Standards for reporting qualitative research: a synthesis of recommendations.** *Academic Medicine*, Vol. 89, No. 9 / Sept 2014 DOI: 10.1097/ACM.0000000000000388	P26/1-27, P27/1-27, P28/1-13.

For peer review only

For peer review only - http://bmjopen.bmj.com/site/about/guidelines.xhtml

BMJ Open

Barriers to implementation of enhanced recovery after surgery (ERAS) by a multidisciplinary team in China: a multicentre qualitative study

Journal:	*BMJ Open*
Manuscript ID	bmjopen-2021-053687.R1
Article Type:	Original research
Date Submitted by the Author:	22-Nov-2021
Complete List of Authors:	Wang, Dan; Sichuan University, West China School of Nursing<br>Liu, Zhenmi; Sichuan University, West China School of Public Health and West China Fourth Hospital<br>Zhou, Jing; The Second People's Hospital of Chengdu<br>Yang, Jie; Sichuan University West China Hospital, Department of Gastrointestinal Surgery<br>Chen, Xinrong; Sichuan University, West China School of Nursing<br>Chang, Chengting; Sichuan University, West China School of Nursing<br>Liu, Changqing; Sichuan University, West China School of Nursing<br>Li, Ka; Sichuan University, West China School of Nursing<br>Hu, Jiankun; Sichuan University West China Hospital, Department of Gastrointestinal Surgery
**Primary Subject Heading**:	Surgery
Secondary Subject Heading:	Surgery, Nursing
Keywords:	QUALITATIVE RESEARCH, Organisation of health services < HEALTH SERVICES ADMINISTRATION & MANAGEMENT, Quality in health care < HEALTH SERVICES ADMINISTRATION & MANAGEMENT

SCHOLARONE™
Manuscripts

I, the Submitting Author has the right to grant and does grant on behalf of all authors of the Work (as defined in the below author licence), an exclusive licence and/or a non-exclusive licence for contributions from authors who are: i) UK Crown employees; ii) where BMJ has agreed a CC-BY licence shall apply, and/or iii) in accordance with the terms applicable for US Federal Government officers or employees acting as part of their official duties; on a worldwide, perpetual, irrevocable, royalty-free basis to BMJ Publishing Group Ltd ("BMJ") its licensees and where the relevant Journal is co-owned by BMJ to the co-owners of the Journal, to publish the Work in this journal and any other BMJ products and to exploit all rights, as set out in our licence.

The Submitting Author accepts and understands that any supply made under these terms is made by BMJ to the Submitting Author unless you are acting as an employee on behalf of your employer or a postgraduate student of an affiliated institution which is paying any applicable article publishing charge ("APC") for Open Access articles. Where the Submitting Author wishes to make the Work available on an Open Access basis (and intends to pay the relevant APC), the terms of reuse of such Open Access shall be governed by a Creative Commons licence – details of these licences and which Creative Commons licence will apply to this Work are set out in our licence referred to above.

Other than as permitted in any relevant BMJ Author's Self Archiving Policies, I confirm this Work has not been accepted for publication elsewhere, is not being considered for publication elsewhere and does not duplicate material already published. I confirm all authors consent to publication of this Work and authorise the granting of this licence.

For peer review only

Barriers to implementation of enhanced recovery after surgery (ERAS) by a multidisciplinary team in China: a multicentre qualitative study

Correspondence to Professor Ka Li; lika127@126.com; Dr Jiankun Hu; hujkwch@126.com

Dan Wang,[1] Zhenmi Liu,[2] Jing Zhou,[3] Jie Yang,[4] Xinrong Chen,[1] Chengting Chang,[1] Changqing Liu,[1] Ka Li,[1] Jiankun Hu[4]

Affiliation

1. West China School of Nursing, West China Hospital, Sichuan University, Chengdu, China.

2. West China School of Public Health, West China Hospital, Sichuan University, Chengdu, China.

3. The Second People's Hospital of Chengdu, Chengdu, China.

4. Department of Gastrointestinal Surgery, West China Hospital, Sichuan University, Chengdu, China

Word count 4530 words.

Keywords Enhanced recovery after surgery (ERAS); Multidisciplinary team (MDT); Qualitative study; SPO model.

For peer review only

ABSTRACT

Objective To explore the attitudes and barriers encountered in the implementation of enhanced recovery after surgery (ERAS) in China from the perspective of multidisciplinary team members.

Design Based on Donabedian's structure-process-outcome (SPO) model, a multicentre qualitative study using semistructured interviews was conducted.

Setting From September 2020 to December 2020, the participants of this study were interviewed from six tertiary hospitals in Sichuan Province (n=3), Jiangsu Province (n=2) and Guangxi Province (n=1) in China.

Participants A total of 42 members, including surgeons (n=11), anaesthesiologists (n=10), surgical nurses(n=14) and dietitians(n=7) were interviewed.

Results Multidisciplinary team (MDT) members still face many barriers during the process of implementing ERAS. Eight main themes are described around the barriers in the implementation of ERAS. Themes in the structure dimension are: (1) shortage of medical resources, (2) lack of policy support, (3) outdated concepts. Themes in the process dimension are: (1) poor doctor-patient collaboration, (2) poor communication and collaboration among MDT members, (3) lack of individualized management. Themes in the outcome dimension are: (1) low compliance, (2) high medical costs. The current implementation of ERAS is still based on ideas more than reality.

Conclusions In general, barriers to ERAS implementation are broad. Identifying key elements of problems in the application and promotion of ERAS from the perspective of the multidisciplinary team would provide a starting point for future quality

For peer review only

improvement of ERAS, enhance the clinical effect of ERAS and increase formalized ERAS utilization in China.

Strengths and limitations of this study

- This study is the first multicentre qualitative study of ERAS in China that explored the barriers encountered in the implementation and promotion of ERAS from the perspective of multidisciplinary team members.
- This study included dietitians as one of the research objects to analyse the views and opinions of dietitians about the implementation of ERAS.
- This study used the SPO model as theoretical support to analyse the barriers in the application process of ERAS from a multidimensional perspective.
- Convenience sampling could lead to potential sampling bias.
- There were potential hints and prejudices in questions raised and interviewer's explanations.

INTRODUCTION

The surgical stress response associated with major surgery describes fundamental metabolic changes that lead to increased catabolism, immunosuppression, free radical production, and hypercoagulable states.[1] Enhanced recovery after surgery (ERAS) programs were introduced and implemented by Danish surgeon H. Kehlet in the 1990s.[1] The core concept of ERAS is to apply a series of evidence-based, innovative and optimized perioperative measures through multidisciplinary teamwork, such as prehabilitation, minimally invasive surgery, removal of invasive lines, multimodal analgesia, early mobilization and early oral feeding, to reduce ineffective and unhelpful medical interventions and reduce the incidence of postoperative stress and complications, thereby speeding up the recovery and adaptation of patients after surgery.[2-5] Based on these advantages, the ERAS program has been adopted internationally and applied widely in the field of surgery.

For peer review only - http://bmjopen.bmj.com/site/about/guidelines.xhtml

In China, the ERAS program was first proposed and implemented by Professor Jieshou Li in many fields of general surgery, such as stomach and colorectal surgery, and achieved a good curative effect for patients.[6 7] Subsequently, ERAS programs have been popularized and applied in tertiary hospitals (the highest level) in various provinces of China for more than 10 years. Through a large number of randomized controlled trials (RCTs), it has been proven that the implementation of ERAS can effectively reduce the pain of patients, hospitalization costs and length of stay in the hospital without increasing the 30-day readmission rate and complications.[8-11] At present, the ERAS program has been successfully applied to general,[12] orthopaedic,[10] urological[11] and other operations,[13] and many expert consensuses have been developed for each specialty, marking the maturity of the application of ERAS in China.

There is currently sufficient evidence to support the hypothesis that the implementation of ERAS can indeed effectively improve the efficiency of the use of health service resources[14] and improve patient satisfaction with the diagnosis and treatment results.[15 16] However, there are variations in the implementation of ERAS for different regions, hospitals at different levels and experts in different disciplines, [17-19] which limits the application and popularization of ERAS.

The ERAS pathway is carried out in the form of multidisciplinary cooperation, including multiple modes of surgery, anaesthesia, nursing and perioperative management to ensure the safety and effectiveness of patients during the perioperative period. [4] Previous studies have proven that multidisciplinary teams (MDTs) are an important guarantee for ERAS, and the effective cooperation of multidisciplinary teams is a key positive factor in promoting the application and advancement of ERAS.[20] Overall, the establishment of a patient-centred MDT is an inevitable trend in the development of ERAS. However, the construction and management of ERAS-MDT is still in the exploratory stage, and multidisciplinary members still face many barriers and challenges in the various processes of implementing ERAS.[21 22] Therefore, a targeted analysis of various barriers and a quality management plan are crucial to the

For peer review only - http://bmjopen.bmj.com/site/about/guidelines.xhtml

construction and development of ERAS-MDT, which is expected to speed up the implementation of ERAS in China.

Previous studies have found many barriers to interprofessional cooperation among ERAS-MDTs, such as a lack of personnel, resistance to changes in traditional surgical concepts, poor patient compliance and collaboration, and a lack of unified management and operative plans for MDTs.[22-25] However, the current qualitative research mainly focused on surgeons, nurses, and anaesthesiologists.[25] There are only limited data on the barriers encountered by dietitians in the implementation of ERAS,[26] warranting further study.

In this study, based on Donabedian's structure-process-outcome health care quality model (SPO model), we presented the results of a qualitative study that aimed to gain an in-depth understanding of the views and opinions of multidisciplinary members in different regions on the implementation and promotion of the ERAS program and to explore the barriers that affect the effective implementation of ERAS, so as to provide a basis for the establishment of the quality evaluation system of ERAS, which can contribute to the development of ERAS programs in China.

METHODS

Based on the SPO model, we conducted a multicentre qualitative interview study to explore the experience of surgeons, anaesthesiologists, nurses and dietitians in implementing the ERAS program from the three aspects of structure, process and outcome and to analyse the barriers that affect the promotion and application of ERAS programs.

Theoretical Framework

The SPO model proposed by Donabedian considers both internal and external organizational factors. Internally, it covers an organization's structure (S), process (P), and outcome (O), and their interactions. On the other hand, external factors, such as

For peer review only - http://bmjopen.bmj.com/site/about/guidelines.xhtml

health and social factors are also combined in this model.[27 28] The SPO model also has theoretical underpinnings in that good structure should promote good process and good process should promote good outcomes, which is conducive to guiding the effective improvement of medical quality.[29] Therefore, the approach of Donabedian is valuable in conceptualizing and organizing medical quality evaluation,[30] and has been widely adopted in the research of ERAS quality evaluation.[31] In this qualitative interview study, the SPO model was used to analyze the barriers of structure, process, and outcome that potentially affect the implementation of ERAS programs. The ‘structure’ refers to the methods of resource allocation and the efficiency of resource utilization in medical services, such as the adequacy of facilities and equipment, sufficient staff and financial resources, and complete management policies. ‘Process’ refers to the specific execution quantity and quality of each link in the implementation of medical activities according to the plan, such as the execution rate and execution quality of each ERAS program. The ‘outcome’ refers to the final result after the end of medical services, such as patient satisfaction, rehabilitation effect and medical cost. [27 28]

Study design and setting

Data collection used a qualitative descriptive approach and semistructured interviews. The ERAS program was mainly implemented in tertiary public hospitals in China, and convenience sampling was used to select hospitals in our study. The criteria for selecting the hospitals are shown in Figure 1. Participants in our study were interviewed from six tertiary hospitals in Sichuan Province (n=3), Jiangsu Province (n=2) and Guangxi Province (n=1). The ERAS implementation process, year, surgical field, compliance, and institutional protocol of the hospitals were shown in Supplementary file 1. We also signed the multi-center institutional protocol, and the institutional protocol was the Chinese version (Supplementary file 2).

For peer review only - http://bmjopen.bmj.com/site/about/guidelines.xhtml

Sample

Purposive sampling was used to provide a diverse range of ages, genders, occupational classification and locations (Table 1).[32] 42 members were included in the analysis, including 11 surgeons, 10 anaesthesiologists, 14 surgical nurses and 7 dietitians; 9 were excluded: 2 for poor quality of recording, 2 for schedule clash, 5 for data saturation. The inclusion criteria of the ERAS-MDT members were as follows: (1) Experience in ERAS implementation; (2) More than 10 years of working experience; and (3) Gave informed consent and volunteered to participate in the study. Participants who could not express their ideas accurately and fully were excluded.

According to the inclusion and exclusion criteria, the relevant persons-in-charge of each hospital selected the research subjects who met the purpose of this study and sent a message to invite these research subjects to participate in this study. If they agreed to participate and share their experience and views on the implementation of ERAS, the first author (Dan Wang) contacted them by telephone and registered them to participate in this study with written informed consent.

Data collection

Interviewees were categorized by location of work site (i.e., Sichuan, Jiangsu or Guangxi Province) and role (i.e., surgeon, anaesthesiologists, surgical nurse and dietitian). The participants were interviewed individually in the period from September to December 2020. Participants in Chengdu, Sichuan Province, conducted face-to-face interviews in a quiet room at their hospitals. Participants from other provinces were interviewed by telephone. The principal investigator (Dan Wang) and research assistant (Jing Zhou, Xinrong Chen and Chengting Chang) conducted the interviews together. All interviewers has completed training in interview. None of the participants had a close personal relationship with the interviewers, and all participants agreed to be audiotaped. According to the SPO model, a semi-structured topic guide was used for the interviews, as follows:

For peer review only

For peer review only - http://bmjopen.bmj.com/site/about/guidelines.xhtml

- Judging from the current hospital policy management and basic resources, what do you think are the key factors that hinder the implementation of ERAS?
- During the perioperative period, which measures recommended by the ERAS guidelines have barriers and difficulties in implementation? What factors caused these barriers?
- What difficulties are faced by multidisciplinary teams in the process of cooperating to implement ERAS programs?
- What barriers do patients and their families cause to the implementation of ERAS programs?
- From your professional perspective, do you think what measures should be taken to improve the implementation effect of ERAS programs?

During the interview, we flexibly asked different questions based on the respondent's answers and the semi-structured nature of the interviews allowed participants to explore other topics which they considered relevant. Meanwhile, interviewers promptly confirmed the vague information provided by the interviewees. The average interview time was 21.84 minutes. At the end of interviews, the researcher made a brief summary to determine whether there was any missing or additional information and asked the interviewees to fill in the general situation form. The researcher undertook reflection after each interview to check whether the interview process was inappropriate or needed to be improved and to confirm whether the data had reached saturation and whether the interview process needed to continue. This work adheres to Standards for Reporting Qualitative Research (SRQR).

Data analysis

The recorded interview was transcribed verbatim within 24 hours by member of the research team. Transcribed scripts were assigned a unique code in the order of interviews, and names were removed to align with confidentiality. Data analysis followed an thematic approach of induction and explanation,[33] applying principles of

For peer review only - http://bmjopen.bmj.com/site/about/guidelines.xhtml

constant comparison to analyze differences across cases.[34] Main concepts and themes within the data were identified through a combination of open coding and thematic analysis.[35 36] The research team repeated line-by-line reading of the transcripts. Following familiarization, the research team manually did open coding, drawing on their rich clinical experiences, and recorded these codes on the margin of the printed transcript.[37] These codes were subsequently grouped according to the SPO model. Through clustering and integration of codes, themes of each dimension were determined. Themes were considered for inclusion in this report if they were prominent. Prominent themes were further explored in context through in-depth reading and refined until saturation was reached.[38] To ensure reliability, the research team met regularly to review the coded data, verify its relevance to main themes, discuss the interpretations and agree on any new theme which were required. Data collection ceased when no new codes or themes were identified, which meant data saturation. Data saturation was considered to be the point at which coded data from new interviews were only added to existing themes and no new themes were developed.[39] This study finally identified eight themes.

Ethical approval

This study was approved by the Ethics Committee of Biomedical Research (West China Hospital of Sichuan University) (No.2020-1038). Data were treated confidentially. Each participant was assigned a code before data analysis (e.g., Interview 2, Anaesthesia, Male), and therefore, personal identification was removed.

Patient and public involvement

No patients were involved in this study. The public were not involved in the development of the research questions, research design or outcome measures.

RESULTS

For peer review only - http://bmjopen.bmj.com/site/about/guidelines.xhtml

Structure

1. Shortage of medical resources

All participants described the shortage of medical resources as the main barrier to their implementation of the ERAS program, and the most common description was the pressure and challenges caused by the shortage of human resources. Due to the application of ERAS, many measures needed to be implemented, and to shorten the average length of stay and improve the bed turnover rate, doctors and nurses must take care of more patients with limited resources and time. (table 2, Q1-Q2). Many participants described that the workload of doctors and nurses has increased, but hospital leaders have not increased their corresponding salaries and bonuses, which could not motivate the medical staff to implement the ERAS program (table 2, Q3). Surgeons and anaesthesiologists in 3 hospitals all mentioned that the operating room of the hospital was not sufficient to provide more equipment and space to satisfy all patients who could carry out the ERAS program (table 2, Q4-Q5).

2. Lack of policy support

At the hospital level, many hospitals have not formulated and introduced corresponding incentive policies and measures to guide department leaders and medical staff to actively implement ERAS, so many medical staff do not generate internal driving forces and are unwilling to implement ERAS programs (table 2, Q6). Some participants believed that the ERAS program promoted by tertiary hospitals is generally not implemented in basic-level hospitals, and the disconnection of concepts cannot ensure the smooth implementation of ERAS in the entire treatment process of patients (table 2, Q7). In addition, there is no formal training course or mechanism for the ERAS program in China. Medical staff only learn ERAS through meetings, discussions in the department, and exchanges with other hospitals. This informal training does not have a unified ERAS implementation process, which leads to great differences in the

For peer review only - http://bmjopen.bmj.com/site/about/guidelines.xhtml

implementation of ERAS in various hospitals, and it is impossible to compare and analyse the implementation effects of multicentre hospitals (table 2, Q8).

3. Outdated concepts

Doctors are reluctant to try new concepts mainly because they are worried about the safety of patients. The doctor-patient relationship is more subtle in China. If doctors encounter problems in the process of implementing ERAS, they will face greater work pressure and professional crises. Doctors take patient safety as their first priority in any medical measures they take, so they dare not try ERAS lightly (table 2, Q9-Q10).

Process

4. Poor doctor-patient collaboration

Participants also mentioned the importance of doctor-patient collaboration and described situations that hinder the development of some ERAS programs due to the unsatisfactory process of doctor-patient collaboration (Table 3). In terms of preoperative education, doctors believed that because patients have a low degree of understanding and significant medical knowledge gaps, if the information conveyed to patients is not sufficient and clear, the patient will not be able to accurately understand the specific connotations of the doctor's instructions, which will lead to deviation in the process of cooperation with patients and hinder the normal development of some ERAS programs (Table 3, Q1-Q2).

The medical staff mainly mentioned that patients would mistakenly remember the time of preoperative fasting, which would affect ERAS implementation and delay the operation time. Some patients who have undergone traditional surgery will question the ERAS concept of preoperative fasting, worrying about accidents during the operation (Table 3, Q3). In terms of early oral feeding, the nurses mentioned that they must personally supervise the patients to make them eat on time. If the supervision is not timely, the patient will delay eating. Some patients will refuse to eat early if they feel

For peer review only - http://bmjopen.bmj.com/site/about/guidelines.xhtml

unwell, or if they feel unwell after eating, they will be dissatisfied with the quality of ERAS (Table 3, Q4-Q5).

Participants said that the traditional concept of patients was that they needed to lie in bed for a long time after surgery because they worried that early mobilization would affect wound healing and aggravate pain (Table 3, Q6-Q7). All dietitians mentioned that due to the shortened length of hospital stay and the lack of a complete postoperative follow-up mechanism, they could only obtain some subjective indicators through the phone, which made them unable to objectively and accurately evaluate the implementation effect of the ERAS nutrition program. The doctors believed that due to the shortened length of hospital stay, the patient's later treatment cannot be effectively guaranteed, which would increase the dissatisfaction of the patient. If postoperative complications occurred and the patient was re-admitted to the hospital for treatment, it would intensify the doctor-patient contradiction (Table 3, Q8-Q9).

Participants emphasized that patients' trust and adequate education were the key factors to improve the effect of doctor-patient collaboration. Some staff believed that patients’ trust in doctors’ diagnostic and treatment skills was the cornerstone of the implementation of ERAS. In addition, adequate education could promote patients' understanding of the diagnosis and treatment plan and further improve patient satisfaction and compliance (table 4, Q1).

5. Poor communication and collaboration among multidisciplinary members

Participants mentioned that the main difficulty affecting the implementation of the ERAS program was the communication and collaboration between multidisciplinary teams, which would not only have a negative impact on the treatment of patients but also bring challenges to the smooth implementation of ERAS (table 4, Q2-Q3). Participants described that the continuous and effective communication and collaboration of the multidisciplinary team throughout the perioperative period played a vital role in the smooth progress of ERAS. They also emphasized that different

For peer review only - http://bmjopen.bmj.com/site/about/guidelines.xhtml

disciplines must reach a consensus on the ERAS program and jointly discuss and formulate a series of standardized implementation standards and procedures to improve the overall efficiency of collaboration between ERAS-MDT and increase the success rate of ERAS implementation (table 4, Q4).

6. Lack of individualized management

Surgeons and anaesthesiologists mentioned that there was a general lack of personalized perioperative management programs for ERAS. Due to great individual differences of patients, medical staff should fully consider the individual characteristics of each patient's condition when implementing the ERAS program and adopt individualized management plans. Otherwise, safety problems may occur for some patients with a complicated aetiology and many serious comorbidities, which will affect the implementation effect of ERAS (table 4, Q5- Q6).

Outcome

7. Low compliance

All participants believed that the compliance of patients and families was one of the important factors affecting the effectiveness of ERAS and that good compliance was an important prerequisite for ensuring the safety and effectiveness of treatment. Participants mentioned that the poor compliance of some patients and their families would reduce the effectiveness of ERAS and even cause a waste of medical resources (table 5, Q1- Q2).

8. High cost

Anaesthetists mentioned that to save money, patients with poor economic conditions would choose to endure pain and refuse to use nerve blocks or analgesic pumps. Nutritionists believed that due to the high price of commercial nutritional preparations and the fact that medical insurance does not reimburse this part of the cost, some patients would refuse to use nutritional preparations, leading to the lack of essential

For peer review only - http://bmjopen.bmj.com/site/about/guidelines.xhtml

nutrients in the diet, which affects the postoperative nutritional treatment (table 5, Q3-Q4).

DISCUSSION

Based on SPO theory, we conducted a qualitative study on multidisciplinary ERAS members of six tertiary hospitals in three provinces in China. We coded and refined the theme from the three dimensions of structure-process-outcome and summarized eight main barriers. To our knowledge, this is the first multicentre qualitative article that analyses ERAS application barriers from the perspective of multidisciplinary members in China.

Although medical staff implement the ERAS programs under the guidance of the ERAS guidelines, we found that the audit system has not been established or perfected, many feedback problems cannot be effectively resolved, causing MDT members to face many barriers in the process of implementing ERAS. On the structural dimension, limited medical resources were not conducive to improving the quality of medical services of the ERAS program. Similar to previous evidence,[21 25] we found that in the reality of a heavy disease burden and overloaded work intensity, the shortage of doctors, nurses and other medical staff has led to poor completion quality or failure to implement some ERAS programs, which has become an important factor hindering the sustained, stable and rapid development of ERAS. New findings in our study were that due to the funding gap between government financial appropriations and the actual needs of hospitals, and the government and hospitals had insufficient special financial support for medical and health resources of ERAS, resulting in insufficient reserves of beds, equipment, medicines and other materials. In addition, MDT members who implemented ERAS did not receive increased salaries or bonuses, which led to the slow application of ERAS in clinical departments and did not promote a large-scale development trend. We recommend that hospital leaders and managers should pay more attention to optimizing the complex process of ERAS, reducing unnecessary work,

For peer review only - http://bmjopen.bmj.com/site/about/guidelines.xhtml

increasing funding for ERAS application, and rewarding MDT members who implement ERAS, so as to actively promote the implementation and development of ERAS.

The medical treatment combination was one of China's important measures to deepen the reform of public hospitals, aiming to build a scientific and reasonable medical service system through the alliance and resource sharing of medical and health institutions at different levels.[40] However, we found that the new concepts and new technologies of the ERAS program in primary hospitals are still in the exploratory stage, failing to keep up with the implementation of ERAS in tertiary hospitals, resulting in patients still receiving traditional treatments during recuperation in primary hospitals after discharge, which makes the application of ERAS appear inconsistent. In addition, unlike other studies[22 25], we found that the absence of a unified standardized ERAS course training mechanism in China has led to deviations in the understanding and mastery of ERAS among multidisciplinary members. Previous studies have proven that the establishment of standardized ERAS training courses would provide more scientific and standardized knowledge education and skills training, which would help medical staff to change their concepts and achieve better consistency.[41] We suggested that policies should be issued at the national level to vigorously support and promote the development of ERAS, build and promote a standardized training system for ERAS, improve the awareness and acceptance of ERAS among medical staff at all levels of medical units, update and unify new concepts in a timely manner, promote the construction of the ERAS medical consortium and continue to improve supporting policies to strengthen the service capabilities of medical and health institutions.

In the process dimension, the lack of doctor-patient collaboration was one of the barriers to the implementation of ERAS, which could appear in all aspects of the perioperative period. For example, patients are reluctant to follow the advice given by medical staff to engage in early activities after surgery. The reasons are related to patients' poor understanding and acceptance of ERAS, as well as ineffective

For peer review only - http://bmjopen.bmj.com/site/about/guidelines.xhtml

communication between doctors and patients. Studies have confirmed that good doctor-patient collaboration is an important factor in promoting the effective, safe and efficient operation of the healthcare system.[42] Our study also showed that patients' high trust in medical staff and adequate education by medical staff are the key factors to improving doctor-patient collaboration. Medical staff need to spend enough time providing diagnostic and treatment services for patients and clearly explain their condition to patients, which is a favourable factor for enhancing the collaboration between staff and patients. [13] In addition, individualized management of patients can improve the safety and effectiveness of the implementation of ERAS, which can alleviate patients' worries about the concept, measures and effects of ERAS so that they can trust the medical plans adopted by staff and actively participate in their treatment and recovery.

Previous studies have proven that close multidisciplinary cooperation is an inevitable requirement in the development of ERAS.[43] However, similar to previous evidence,[44] our study also demonstrated that the existing ERAS-MDT model still faces many difficulties in clinical practice. The current model lacks a unified and reasonable overall structure and construction plan, and multidisciplinary members have not fully reached a consensus on ERAS. Furthermore, the lack of scientific management of the implementation path and collaborative operation of ERAS-MDT result in the unclear responsibilities and division of labour of ERAS-MDT members. Meanwhile, the communication, coordination, and perioperative connection of various links need to be further optimized. Therefore, We suggest that taking the patient as the centre and relying on a multidisciplinary team to formulate a standardized, individualized, and continuous comprehensive diagnosis and treatment plan is essential for the implementation of ERAS on the process dimension.

On the outcome dimension, participants agreed that poor compliance of patients and families would cause the quality of ERAS implementation to decline. Previous studies have shown that increased compliance with ERAS programs would lead to better treatment outcomes and may have long-term benefits for survival.[23] Therefore, the

For peer review only

For peer review only - http://bmjopen.bmj.com/site/about/guidelines.xhtml

compliance of patients and families to all ERAS programs is an important indicator to ensure the implementation effect of ERAS. Our study demonstrated that high costs were one of the main reasons for the poor compliance of patients and families. With the continuous process of medical reform, the Social Basic Medical Insurance (SBMI) has covered more than 97% of residents in China and has gradually become an important way to solve the problem of expensive medical treatment for residents.[45] However, it is not free medical care and patients still have to pay part of the cost, which would make some patients with poor financial conditions resist some self-financed treatment measures, such as not eating nutritional preparations and refusing analgesic pumps. We suggest that the government and hospitals should actively introduce plans to reduce medical costs and drug expenses, and further expand the items and scope of medical insurance reimbursement, thereby improving the compliance of the ERAS project.

With the SPO model as a theoretical support, this study is an innovative multicentre qualitative study of ERAS in China that explores the barriers encountered in the implementation and promotion of ERAS from the perspective of multidisciplinary team members. In addition, this study included dietitians as one of the research objects to understand and analyse the views and opinions of dietitians on the implementation of ERAS to compensate for the limitations of previous studies. This study also has limitations. First, the survey results were limited by the self-reporting nature of the interviews. We tried our best to collect different levels of information in terms of age, gender, working years, professional title, and working region to compensate for the limitations of personal cognition. Second, there were potential hints and prejudices in both the questions raised and the guidance and explanations of the interviewer. We selected assistants who did not know ERAS to participate in the interview to avoid personal experience affecting the views of the research subjects. Finally, this study did not interview members of all disciplines involved in the implementation of ERAS, such as pharmacists and rehabilitation doctors. In addition, the attitudes and opinions of health system leaders and patients play a vital role in the application and development

For peer review only - http://bmjopen.bmj.com/site/about/guidelines.xhtml

of ERAS. In the future, we will further expand the types of research objects and improve the research content.

Conclusions

This study analysed the barriers to the implementation of the ERAS program in the structure-process-outcome dimensions from the perspective of multidisciplinary members. Although a large amount of data has shown the advantages of the ERAS program, this study reveals the fact that the implementation of ERAS still faces many obstacles, and there is need to enrich supporting resources, optimize processes to improve implementation effects in China. This study is a multicentre qualitative study, and all hospitals included belong to the southern region of China. Therefore, our results can partially reflect the application status of ERAS in the southern region. In general, the current implementation of ERAS was still based on ideas more than reality. This study has identified common barriers for MDT members to use ERAS in the three provinces of China, and have provided pragmatic solutions to each obstacle. More quality improvement research, evaluation and audit will be needed to improve the application effect of ERAS in the future. We hope that this study provides a starting point for future quality improvement of ERAS, enhance the clinical effect of ERAS and increase formalized ERAS utilization in China.

Footnotes

Contributors KL planned the study. DW was principal investigator, collected, analysed and interpreted the data, and wrote the manuscript. KL, DW and ZML designed the study, developed the methods. ZML, CQL and JKH reviewed/edited the manuscript. JZ, CTC, XRC collected, analysed and checked the data. JY helped draft the paper, provided critical reviews and intellectual content. All authors have seen and

For peer review only

For peer review only - http://bmjopen.bmj.com/site/about/guidelines.xhtml

approved the final version of the manuscript. DW is the guarantor. KL, ZML and JKH are on the supervisory team for DW PhD study.

Finding This work was supported by a program of National Natural Science Foundation of China (71974135). The funders had no role in the study design, data collection and analysis, decision to publish, or preparation of the manuscript.

Competing interests None declared.

Data availability statement Data are available upon reasonable request. The search strategies are available on request to the corresponding author.

Patient consent for publication Not required.

Ethics approval Ethical approval for this study was granted by the Ethics Committee of Biomedical Research (West China Hospital of Sichuan University) (No.2020-1038). Data was treated confidentially.

Provenance and peer review Not commissioned; externally peer reviewed.

REFERENCES

1. Kehlet H. Multimodal approach to control postoperative pathophysiology and rehabilitation. *Br J Anaesth* 1997;78:606-617.
2. Nelson G, Bakkum-Gamez J, Kalogera E, *et al*. Guidelines for perioperative care in gynecologic/oncology: Enhanced Recovery After Surgery (ERAS) Society recommendations-2019 update. *Int J Gynecol Cancer* 2019;29:651-668.
3. Wilmore DW, Kehlet H. Management of patients in fast track surgery. *Br Med J* 2001;322:473–476.

For peer review only

For peer review only - http://bmjopen.bmj.com/site/about/guidelines.xhtml

4. Ljungqvist O, Scott M, Fearon KC. Enhanced recovery after surgery: a review. *JAMA Surg* 2017;152:292-298.

5. Department of Health. Enhanced recovery partnership programme project report. 2011 https://www.gov.uk/government/publications/ enhanced-recovery-partnership-programme (accessed 1 Feb 2017).

6. Zhao J, Hu J, Jiang Z, *et al*. Outcome of Discharge Within 72 Hours of Robotic Gastrectomy Using Enhanced Recovery After Surgery Programs. *J Laparoendosc Adv Surg Tech A* 2018;28:1279-1286.

7. Liu XX, Pan HF, Jiang ZW, *et al*. "Fast-track" and "Minimally Invasive" Surgery for Gastric Cancer. *Chin Med J (Engl)* 2016;129:2294-2300.

8. Kjølhede P, Bergdahl O, Borendal Wodlin N, *et al*. Effect of intrathecal morphine and epidural analgesia on postoperative recovery after abdominal surgery for gynecologic malignancy: an open-label randomised trial. *BMJ Open* 2019;9:e024484.

9. Liang X, Ying H, Wang H, *et al*. Enhanced recovery care versus traditional care after laparoscopic liver resections: a randomized controlled trial. *Surg Endosc* 2018;32:2746-2757.

10. Cui H, Sun Z, Ruan J, *et al*. Effect of enhanced recovery after surgery (ERAS) pathway on the postoperative outcomes of elbow arthrolysis: A randomized controlled trial. *Int J Surg* 2019;68:78-84.

11. Lin T, Li K, Liu H, *et al*. Enhanced recovery after surgery for radical cystectomy with ileal urinary diversion: a multi-institutional, randomized, controlled trial from the Chinese bladder cancer consortium. *World J Urol* 2018;36:41-50.

12. Xu X, Wang Y, Feng T, *et al*. Nonstrict and individual enhanced recovery after surgery (ERAS) in partial hepatectomy. *Springerplus* 2016;5:2011.

13. Liu B, Liu S, Wang Y, *et al*. Neurosurgical enhanced recovery after surgery (ERAS) programme for elective craniotomies: are patients satisfied with their experiences? A quantitative and qualitative analysis. *BMJ Open* 2019;9:e028706.

For peer review only

For peer review only - http://bmjopen.bmj.com/site/about/guidelines.xhtml

14. Yamagata Y, Yoshikawa T, Yura M, *et al*. Current status of the "enhanced recovery after surgery" program in gastric cancer surgery. *Ann Gastroenterol Surg* 2019;3:231-238.

15. Suharwardy S, Carvalho B. Enhanced recovery after surgery for cesarean delivery. *Curr Opin Obstet Gynecol* 2020;32:113-120.

16. Kołodziej T, Maciejewski T, Mendrala K, *et al*. Enhanced recovery after cardiac surgery. *Kardiochir Torakochirurgia Pol* 2019;16:32-36.

17. Rossoni C, Oliveira Magro D, Santos ZC, *et al*. Enhanced Recovery After Surgery (ERAS) protocol in bariatric and metabolic surgery (BMS)-analysis of practices in nutritional aspects from five continents. *Obes Surg* 2020;30:4510-4518.

18. Chen X, Li K, Yang K, *et al*. Effects of preoperative oral single-dose and double-dose carbohydrates on insulin resistance in patients undergoing gastrectomy:a prospective randomized controlled trial. *Clin Nutr* 2021;40:1596-1603.

19. Rattray M, Roberts S, Desbrow B, *et al*. A qualitative exploration of factors influencing medical staffs' decision-making around nutrition prescription after colorectal surgery. *BMC Health Serv Res* 2019;19:178.

20. Paton F , Chambers D , Wilson P , *et al*. Effectiveness and implementation of enhanced recovery after surgery programmes: a rapid evidence synthesis. *BMJ Open* 2014;4:e005015.

21. Pearsall EA, McLeod RS. Enhanced Recovery After Surgery: Implementation Strategies, Barriers and Facilitators. *Surg Clin North Am* 2018;98:1201-1210.

22. Cohen R, Gooberman-Hill R. Staff experiences of enhanced recovery after surgery: systematic review of qualitative studies. *BMJ Open* 2019;9:e022259.

23. Seow-En I, Wu J, Yang LWY, *et al*. Results of a colorectal enhanced recovery after surgery (ERAS) programme and a qualitative analysis of healthcare workers' perspectives. *Asian J Surg* 2021;44:307-312.

For peer review only

For peer review only - http://bmjopen.bmj.com/site/about/guidelines.xhtml

24. Wong DJN, Harris SK, Moonesinghe SR, *et al*. Cancelled operations: a 7-day cohort study of planned adult inpatient surgery in 245 UK National Health Service hospitals. *Br J Anaesth* 2018;121:730-738.

25. Pearsall EA, Meghji Z, Pitzul KB, *et al*. A qualitative study to understand the barriers and enablers in implementing an enhanced recovery after surgery program. *Ann Surg* 2015;261:92-96.

26. Byrnes A, Young A, Mudge A, *et.al*. Exploring practice gaps to improve PERIoperativE Nutrition CarE (EXPERIENCE Study): a qualitative analysis of barriers to implementation of evidence-based practice guidelines. *Eur J Clin Nutr* 2019;73:94-101.

27. Donabedian A. Evaluating the quality of medical care. 1966. *Milbank Q* 2005;83:691-729.

28. Donabedian A. The quality of care. How can it be assessed? *JAMA* 1988;260:1743-8.

29. Castle NG, Ferguson JC. What is nursing home quality and how is it measured?. *Gerontologist* 2010;50:426-442.

30. Cherry RL. Agents of nursing home quality of care: ombudsmen and staff ratios revisited. *Gerontologist* 1991;31:302-308.

31. Shah R, Diaz A, Tripepi M, *et al*. Quality Versus Costs Related to Gastrointestinal Surgery: Disentangling the Value Proposition. *J Gastrointest Surg* 2020;24:2874-2883.

32. Mays N, Pope C. Rigour and qualitative research. *BMJ* 1995;311:109–12.

33. Clarke V, Braun V, Hayfield N. Thematic analysis. In: Smith JA, ed. *Qualitative psychology: a practical guide to research methods.* 3rd edition. Sage Publications, 2015.

34. Fram S. The constant comparative analysis method outside of Grounded theory. *Qual Rep* 2013;18:1–25.

35. Boyatzis R. *Transforming qualitative information*. London: Sage, 1998.

For peer review only

For peer review only - http://bmjopen.bmj.com/site/about/guidelines.xhtml

36. Guest G, MacQueen KM, Namey E. *Introduction to applied thematic analysis, Applied Thematic Analysis*. London: SAGE Publications, 2012.

37. Braun V, Clarke V. Using thematic analysis in psychology. *Qual Res Psychol* 2006;3:77–101.

38. Gramlich L, Nelson G, Nelson A, *et al*. Moving enhanced recovery after surgery from implementation to sustainability across a health system: a qualitative assessment of leadership perspectives. *BMC Health Serv Res* 2020;20:361.

39. Saunders B, Sim J, Kingstone T, *et al*. Saturation in qualitative research: exploring its conceptualization and operationalization. *Qual Quant* 2018;52:1893–907.

40. Wang J, Wang Y, Cai H, *et al*. Analysis of the status quo of the Elderly's demands of medical and elderly care combination in the underdeveloped regions of Western China and its influencing factors: a case study of Lanzhou. *BMC Geriatr* 2020;20:338.

41. Francis NK, Walker T, Carter F, *et al*. Consensus on Training and Implementation of Enhanced Recovery After Surgery: A Delphi Study. *World J Surg* 2018;42:1919-1928.

42. Penm J, MacKinnon NJ, Strakowski SM, *et al*. Minding the Gap: Factors Associated With Primary Care Coordination of Adults in 11 Countries. *Ann Fam Med* 2017;15:113-119.

43. Li L, Jin JY, Min S, *et al*. Compliance with the enhanced recovery after surgery protocol and prognosis after colorectal cancer surgery: a prospective cohort study. *Oncotarget* 2017,8:53531-53541.

44. Lyon A, Solomon MJ, Harrison JD. A qualitative study assessing the barriers to implementation of enhanced recovery after surgery. *World J Surg* 2014;38:1374-1380.

45. Li H, Jiang L. Catastrophic medical insurance in China. *Lancet* 2017;390:1724–5.

For peer review only - http://bmjopen.bmj.com/site/about/guidelines.xhtml

Tables

**Table 1** Characteristics of interviewees	
Mean age (SD, range)	37.36 (SD 5.94; range 28–52)
Characteristic	n(%)
Gender	
Male	20(47.62)
Female	22(52.38)
Occupational classification	
surgeon	11(26.19)
anaesthesiologist	10(23.81)
surgical nurse	14(33.33)
dietitian	7(16.67)
Location	
Sichuan	25(59.52)
Jiangsu	10(23.81)
Guangxi	7(16.67)

Table 2 Illustrative quotes representative of theme 1, theme 2 and theme 3

**Q**	**Illustrative quotes**
1	"since the number of nurses did not increased, the quality of implementation would be affected when the workload was particularly heavy." (Interview 39, Nurse, Female)
2	"...some surgeons thought that getting a nerve block would delay his operation time... Because there were too many patients in China, surgeons had to perform more operations in a limited time..." (Interview 13, Anaesthesia, Male)
3	"...our salary and bonuses did not change after implementing so many ERAS measures." (Interview 25, Dietician, Male)

For peer review only

4	"Not every patient in our department routinely uses equipment such as insulation blankets and infusion fluid warming, and not every patient can be monitored for temperature. "(Interview 14, Anaesthesia, Male)
5	"If we have more operating rooms, our nerve block can be performed in the preparation room in advance, so the whole process will be smoother." (Interview 19, Anaesthesia, Female)
6	"Er, I think hospital leaders can stimulate our enthusiasm for implementing ERAS through various policies. For example, ... the clinical implementation effects and scientific research results of ERAS should be vigorously publicized." (Interview 11, Anaesthesia, Male)
7	"Currently, basic-level hospitals generally do not carry out ERAS programs. This is actually a big problem." (Interview 2, Nurse, Female)
8	"There is currently no standardized ERAS training. If this can be realized in the future, ERAS can be implemented in a more unified and standardized way..." (Interview 35, Anaesthesia, Male)
9	"Many surgeons are based on their own practice standards and are reluctant to accept the various new concepts of ERAS. This is the most critical issue... "(Interview 11, Anaesthesia, Male)
10	"We require local anaesthesia and analgesia for wounds, but surgeons believe that this will lead to liquefaction of the wound and increase the incidence of infection. Although we told them that this will not increase the incidence of wound infection, they are very stubborn, and it is difficult for us to communicate with them. "(Interview 14, Anaesthesia, Male)

Table 3 Illustrative quotes representative of theme 4

Programs	Q	Illustrative quotes
**Preoperative education**	1	"Patients often complained that doctor were too busy, and they could not find doctors if they wanted to consult. So have patients been given enough information by staff in hospitals? "(Interview 32, Surgeon, Male)
	2	"Sometimes we thought we had explained contents clearly to the patient, but in fact he might not truly understand them. "(Interview 27, Nurse, Female)

For peer review only

**Preoperative fasting**	3	"Patients with traditional surgery experience would worry about aspiration, so he would not eat anything. Some patients would forget the exact time of fasting or postpone the prescribed time." (Interview 40, Dietician, Female)
**Early oral feeding**	4	"Patients would not voluntarily go to eat early after surgery, so we must always supervise and remind them. Some patients were reluctant to eat carbohydrate nutrient solution, mainly because they were worried that the gastrointestinal tract would be uncomfortable after taking it. "(Interview 6, Nurse, Female)
	5	"We encouraged patients to eat early after surgery, but if they had gastric bloating after eating, they would question our ERAS." (Interview 30, Surgeon, Male)
**Early mobilization**	6	"The traditional Chinese concept is to lie in bed for a long time after surgery, which is difficult for patients to change." (Interview 20, Anaesthesia, Male)
	7	"We would tell patients who early mobilization could enhanced recovery, but they would question this measure, worrying whether it would make surgical wound dehiscence or exacerbate pain." (Interview 1, Nurse, Female)
**Postoperative follow-up**	8	"We hope to get the evaluation of the nutritional management effect of patients throughout the perioperative period. However, for example, patients with gallstone surgery were only hospitalized for one day, which made it difficult for us to obtain objective indicators for postoperative nutritional assessment." (Interview 23, Dietician, Female)
	9	"The medical care and security that patients received after discharge were not enough. Once complications occurred and patients were re-admitted to the hospital, the doctor-patient conflict would become more intense,

For peer review only

	which would cause many mental pressure on doctors." (Interview 28, Surgeon, Male)

646

Table 4 Illustrative quotes representative of theme 4, theme 5 and theme 6

Q	Illustrative quotes
1	"My personal view is that the patient needs to trust 100% of the diagnosis and treatment made by the medical staff. The second is that medical staff need to do adequate education with patients, such as telling patients about the benefits that new concepts and measures can bring to him, encouraging him to accept changes, and better cooperating with us in various tasks. "(Interview 6, Nurse, Female)
2	"In fact, hospital leaders have been talking about strengthening multidisciplinary cooperation, but now there are very few that truly realize multidisciplinary cooperation, and for teams that do well in multidisciplinary cooperation, they have not been encouraged and played a leading role. "(Interview 14, Anaesthesia, Male)
3	"... multidisciplinary cooperation is the greatest difficulty in promoting ERAS." (Interview 25, Dietician, Male)
4	"Um... I think we should reach a consensus on how to carry out multidisciplinary collaboration in ERAS, and formulate a corresponding process. For example, the division of labour in each discipline is clear, and there is a dedicated person to coordinate the work between multiple disciplines." (Interview 22, Dietician, Female)
5	"Current consensuses of experts were standardized procedures applicable to the general public, there were still individual differences between patients." (Interview 12, Anaesthesia, Male)
6	"In fact, some patients could not perform activities so early after surgery. For example, for elderly patients, the time when they could perform activities may be relatively late. We still have to apply this concept of ERAS in an individualized way." (Interview 23, Dietician, Female)

647

Table 5 Illustrative quotes representative of theme 7 and theme 8

Q	Illustrative quotes
1	"I Occasionally, patients thought that the pain could be tolerated, so they arbitrarily refused to take analgesics prescribed by doctors. However, their pain would increase during activities, which would

For peer review only - http://bmjopen.bmj.com/site/about/guidelines.xhtml

	inevitably affect the implementation of early mobilization and other programs.” (Interview 7, Nurse, Female)
2	“Some patients were unwilling to be discharged early for fear that treatment after discharge would not be guaranteed... patients and families might lie about their illness, which greatly affects the doctor's diagnosis... This way would lead to prolonged hospitalization and waste of resources.” (Interview 8, Surgeon, Male)
3	“... the cost would increase by approximately 500 yuan.... those patients with poor financial conditions would refuse to do the nerve block. In addition, the cost of postoperative analgesia pumps was nearly 1,000 yuan, and there were some patients who would rather bear the pain than spend more money.” (Interview 19, Anaesthesia, Male)
4	“The price of nutritional preparations is very expensive, with a bottle cost of more than 80 yuan. Patients needed to use at least 5 bottles before and after surgery, which would cost more than 400 yuan, and medical insurance was not reimbursed for this cost.” (Interview 21, Dietician, Male)

For peer review only - http://bmjopen.bmj.com/site/about/guidelines.xhtml

➢ A tertiary public hospital.
➢ A general hospital or a specialist hospital.
➢ The hospital has been implementing an ERAS program for at least 5 years.
➢ The hospital agreed to participate in this study.
➢ According to the principle of data saturation, sampling was stopped when there were no new codes or themes.

Figure 1 A summary of the criteria for selecting hospitals

For peer review only

Supplementary file 1 Detailed information on the implementation of ERAS in each center

Hospital	Year	Fields	Compliance
1	2015	Colorectal surgery, Gastrointestinal Surgery, Orthopedic surgery, Hepatobiliary surgery, Urological surgery, Thoracic surgery, Gynecological surgery, Otolaryngology surgery.	≥72%
2	2005	Colorectal surgery, Gastrointestinal Surgery , Orthopedic surgery, Hepatobiliary surgery, Urological surgery, Thoracic surgery, Gynecological surgery.	≥80%
3	2015	Hepatobiliary pancreatic surgery, Gastrointestinal Surgery, Thoracic surgery, Gynecological surgery, Urologic surgery.	≥90%
4	2015	Pancreatectomy, Pancreaticoduodenectomy.	≥80%
5	2012	Hepatobiliary pancreatic surgery, Gastrointestinal Surgery, Thoracic surgery, Urologic surgery.	≥85%
6	2010	Colorectal surgery, Gastrointestinal Surgery , Hepatobiliary surgery, Urological surgery, Thoracic surgery, Gynecological surgery.	≥90%

Each center implements the ERAS pathway in accordance with the ERAS guidelines.

For peer review only

Supplementary file 2 Institutional protocol

多中心研究协议书

项目名称：ERAS 模式下医患协同效果综合评价模型构建与推进路径研究

牵头单位：四川大学华西医院　　研究负责人：李卡

ERAS 模式下医患协同效果综合评价模型构建与推进路径研究各参研中心就该研究相关事宜达成以下协议：

【1】 本单位严格遵守《赫尔辛基》宣言。

【2】 本单位对该研究的研究目的、研究方案、研究核心技术已经充分理解。

【3】 本单位对自身技术力量和工作时间安排经过慎重考虑后自愿参加该项协作研究。

【4】 本单位负责人对所提供的研究数据的真实性和准确性承担全部责任。

【5】 本单位接受和配合本研究学术委员会的监督和管理。

【6】 本单位如有学术不端行为（数据不实、伪造数据、抄袭、剽窃、重复发表、二级发表、一稿两投等），由本分中心负责人承担全部道德和法律责任，而研究主中心和其他分中心免责。

【7】 本单位认可并服从下述知识产权分配制度（主要指发表论文署名排序）的约定，具体如下：

1) 发表论文署名分配策略：（1）论文撰写者+（2）数据分析负责人+（3）有效病例数最多分中心单位的负责人+有效病例数第二多分中心单位的负责人+ ••• +有效病例数最少分中心单位的负责人+（4）其余参研人员（按提供有效病例数多少排序）+（5）本研究牵头 PI 作为通讯作者

2) 若杂志允许，则将论文撰写者、数据分析负责人及有效病例数最多分中心单位的负责人列为共同第一作者或共同通讯作者。

3) 对于期刊论文发表的特殊情况，作者署名尚需根据拟投杂志的要求进行相应调整。如某些 SCI 期刊严格限制论文署名作者数量不超过 8 名，因此仅能将贡献最大前 7-8 名作者列出，其余协作研究者列于文中致谢部分。

4) 为尽量全部体现参研人员的辛勤工作业绩，投稿选择期刊时尽可能避免有作者数量限制的期刊。

5) 期刊选择权及投稿权利在牵头 PI 单位。

6) 本研究相关结果的论文撰写及发表，由牵头 PI 单位执笔。

【8】 针对本研究收集的整体数据，如有参研单位负责人需要二次分析或其他研究目的进行分析时，需得到牵头 PI 单位的同意；参研单位负责人在学会发言需要使用本研究数据时，需注明数据来源并告知牵头 PI 单位。

【9】 参研单位的单中心数据，研究单位负责人有权自行保管，但需遵循隐私保护原则；对于发表的单中心数据结果、形式、内容，相关责任由发表中心负责人自行承担；单中心数据使用时，需告知数据中心并得到准确性认可：但是统计分析的单中心数据需注明来源，避免系统分析时重复纳入。

【10】 各单位数据若合理独立发表，其知识产权归所属分中心所有，不受本研究项目和其他分中心限制，但需在总体研究数据发表之后进行。

【11】 保密原则一：未经该协作研究半数以上分中心同意，任何个人及单位不得向除学术以外任何媒体或个人泄露研究数据及相关信息。

For peer review only - http://bmjopen.bmj.com/site/about/guidelines.xhtml

【12】 研究数据仅供学术研究所用，任何人不得将病例信息用于本研究涉及之外的用途。

【13】 每位论文贡献者均可依照国家及单位相关规定独立使用论文的知识产权，用于晋职、晋级或报奖，无需向其他合作者声明。

【14】 本研究如遇学术争议，依照国内外指南或共识解决，学术委员会有最终裁决权利。

【15】 本研究如遇知识产权争议甚至侵权时，可考虑第三方机构或组织进行磋商调停，或者甚至必要的法律援助。当出现学术不端行为所导致的不良影响或结果时，由当事学术不端研究者或研究单位承担相应道德和法律的责任。

【16】 本协作研究协议书自签字同意之日起执行并产生其协议法律效益。

我已充分理解本协作研究协议书的所有内容，同意（同意/不同意）并自愿参加本研究，遵守所约定的所有内容。

研究分中心：四川大学华西医院

分中心负责人签名：杨婕

日　　期：2020 年 9 月 1 日

牵头单位：四川大学华西医院

负责人签名：[illegible]

日　　期：2020 年 9 月 1 日

For peer review only - http://bmjopen.bmj.com/site/about/guidelines.xhtml

Standards for Reporting Qualitative Research (SRQR)*

http://www.equator-network.org/reporting-guidelines/srqr/

Page/line no(s).

Title and abstract

**Title** - Concise description of the nature and topic of the study Identifying the study as qualitative or indicating the approach (e.g., ethnography, grounded theory) or data collection methods (e.g., interview, focus group) is recommended	P1/1-3.
**Abstract** - Summary of key elements of the study using the abstract format of the intended publication; typically includes background, purpose, methods, results, and conclusions	P2/29-57.

Introduction

**Problem formulation** - Description and significance of the problem/phenomenon studied; review of relevant theory and empirical work; problem statement	P3/71-83, P4/84-111, P5/112-127.
**Purpose or research question** - Purpose of the study and specific objectives or questions	P5/121-127.

Methods

**Qualitative approach and research paradigm** - Qualitative approach (e.g., ethnography, grounded theory, case study, phenomenology, narrative research) and guiding theory if appropriate; identifying the research paradigm (e.g., postpositivist, constructivist/ interpretivist) is also recommended; rationale**	P5/136-139, P6/140-154.
**Researcher characteristics and reflexivity** - Researchers' characteristics that may influence the research, including personal attributes, qualifications/experience, relationship with participants, assumptions, and/or presuppositions; potential or actual interaction between researchers' characteristics and the research questions, approach, methods, results, and/or transferability	P7/188-191.
**Context** - Setting/site and salient contextual factors; rationale**	P6/156-162.
**Sampling strategy** - How and why research participants, documents, or events were selected; criteria for deciding when no further sampling was necessary (e.g., sampling saturation); rationale**	P6/164-167, P7/168-172.
**Ethical issues pertaining to human subjects** - Documentation of approval by an appropriate ethics review board and participant consent, or explanation for lack thereof; other confidentiality and data security issues	P9/237-241.
**Data collection methods** - Types of data collected; details of data collection procedures including (as appropriate) start and stop dates of data collection and analysis, iterative process, triangulation of sources/methods, and modification of procedures in response to evolving study findings; rationale**	P7/180-193, P8/194-202.

For peer review only - http://bmjopen.bmj.com/site/about/guidelines.xhtml

**Data collection instruments and technologies** - Description of instruments (e.g., interview guides, questionnaires) and devices (e.g., audio recorders) used for data collection; if/how the instrument(s) changed over the course of the study	P7/190-193, P8/194-213.
**Units of study** - Number and relevant characteristics of participants, documents, or events included in the study; level of participation (could be reported in results)	P6/156-162; P6/166-167, P7/168-172.
**Data processing** - Methods for processing data prior to and during analysis, including transcription, data entry, data management and security, verification of data integrity, data coding, and anonymization/de-identification of excerpts	P8/216-221, P9/222.
**Data analysis** - Process by which inferences, themes, etc., were identified and developed, including the researchers involved in data analysis; usually references a specific paradigm or approach; rationale**	P9/223-235.
**Techniques to enhance trustworthiness** - Techniques to enhance trustworthiness and credibility of data analysis (e.g., member checking, audit trail, triangulation); rationale**	P9/229-231.

Results/findings

**Synthesis and interpretation** - Main findings (e.g., interpretations, inferences, and themes); might include development of a theory or model, or integration with prior research or theory	P9-P13/247-361.
**Links to empirical data** - Evidence (e.g., quotes, field notes, text excerpts, photographs) to substantiate analytic findings	P24-P28/642-647.

Discussion

**Integration with prior work, implications, transferability, and contribution(s) to the field -** Short summary of main findings; explanation of how findings and conclusions connect to, support, elaborate on, or challenge conclusions of earlier scholarship; discussion of scope of application/generalizability; identification of unique contribution(s) to scholarship in a discipline or field	P13-P17/263-483.
**Limitations** - Trustworthiness and limitations of findings	P17/461-473.

Other

**Conflicts of interest** - Potential sources of influence or perceived influence on study conduct and conclusions; how these were managed	P19/506.
**Funding** - Sources of funding and other support; role of funders in data collection, interpretation, and reporting	P19/502-504.

*The authors created the SRQR by searching the literature to identify guidelines, reporting standards, and critical appraisal criteria for qualitative research; reviewing the reference lists of retrieved sources; and contacting experts to gain feedback. The SRQR aims to improve the transparency of all aspects of qualitative research by providing clear standards for reporting qualitative research.

For peer review only

For peer review only - http://bmjopen.bmj.com/site/about/guidelines.xhtml

**The rationale should briefly discuss the justification for choosing that theory, approach, method, or technique rather than other options available, the assumptions and limitations implicit in those choices, and how those choices influence study conclusions and transferability. As appropriate, the rationale for several items might be discussed together.

**Reference:**	
O'Brien BC, Harris IB, Beckman TJ, Reed DA, Cook DA. **Standards for reporting qualitative research: a synthesis of recommendations.** *Academic Medicine*, Vol. 89, No. 9 / Sept 2014 DOI: 10.1097/ACM.0000000000000388	P19-P23/519-634.

For peer review only

For peer review only - http://bmjopen.bmj.com/site/about/guidelines.xhtml
